# Scc2 Is a Potent Activator of Cohesin’s ATPase that Promotes Loading by Binding Scc1 without Pds5

**DOI:** 10.1016/j.molcel.2018.05.022

**Published:** 2018-06-21

**Authors:** Naomi J. Petela, Thomas G. Gligoris, Jean Metson, Byung-Gil Lee, Menelaos Voulgaris, Bin Hu, Sotaro Kikuchi, Christophe Chapard, Wentao Chen, Eeson Rajendra, Madhusudhan Srinivisan, Hongtao Yu, Jan Löwe, Kim A. Nasmyth

**Affiliations:** 1 Department of Biochemistry, University of Oxford, Oxford OX1 3QU, UK; 2 MRC Laboratory of Molecular Biology, Cambridge CB2 0QH, UK; 3 Department of Molecular Biology and Biotechnology, University of Sheffield, Sheffield S10 2TN, UK; 4 Howard Hughes Medical Institute, Department of Pharmacology, University of Texas Southwestern Medical Center, Dallas, TX 75390, USA

**Keywords:** cohesin, Scc2, Pds5, Scc1, cohesion, ATPase, loading, HAWKs

## Abstract

Cohesin organizes DNA into chromatids, regulates enhancer-promoter interactions, and confers sister chromatid cohesion. Its association with chromosomes is regulated by hook-shaped HEAT repeat proteins that bind Scc1, namely Scc3, Pds5, and Scc2. Unlike Pds5, Scc2 is not a stable cohesin constituent but, as shown here, transiently replaces Pds5. Scc1 mutations that compromise its interaction with Scc2 adversely affect cohesin’s ATPase activity and loading. Moreover, Scc2 mutations that alter how the ATPase responds to DNA abolish loading despite cohesin’s initial association with loading sites. Lastly, Scc2 mutations that permit loading in the absence of Scc4 increase Scc2’s association with chromosomal cohesin and reduce that of Pds5. We suggest that cohesin switches between two states: one with Pds5 bound that is unable to hydrolyze ATP efficiently but is capable of release from chromosomes and another in which Scc2 replaces Pds5 and stimulates ATP hydrolysis necessary for loading and translocation from loading sites.

## Highlights

• Among Hawks, Scc2 is sufficient to activate cohesin’s ATPase in the presence of DNA
• Scc1 mutants that compromise Scc2 binding reduce ATPase activity and cohesin loading
• Scc2 gain-of-function mutations increase its association with chromosomal cohesin
• Increased Scc2 association with chromosomal cohesin correlates with a decrease in Pds5

## Graphical Abstract

**Figure:**
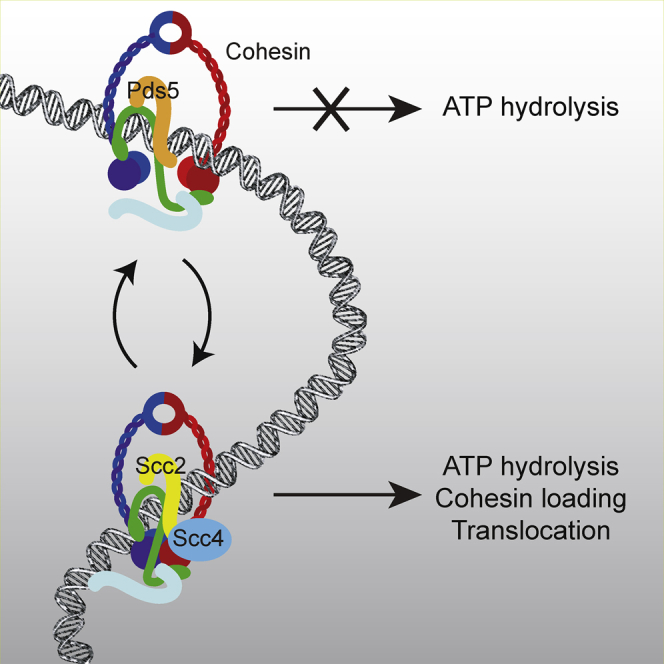

## Teaser

Cohesin switches between two states: one with Pds5 bound that is unable to hydrolyze ATP efficiently but is capable of release from chromosomes and another one in which Scc2 replaces Pds5 and stimulates ATP hydrolysis required for loading and translocation.

## Introduction

How enhancers activate the correct promoters during development, how chromosomal DNAs are woven into chromatids, and how sisters are held together during mitosis are all fundamental questions in chromosome biology. These apparently disparate processes are conferred by a pair of related Smc/kleisin complexes called cohesin and condensin. Both contain a pair of rod-shaped Smc proteins that associate to create V-shape heterodimers (Smc1/3 in cohesin), whose ATPases at their apices are bound by the N- and C-terminal domains of a kleisin subunit (Scc1), forming a huge tripartite ring. In addition to conferring sister chromatid cohesion during G2 and M phases (Nasmyth, 2001), cohesin is involved in the process by which the insulator protein CTCF regulates enhancer-promoter interactions (Fudenberg et al., 2016) and is responsible for creating the topologically associated domains (TADs) detected by HiC (Rao et al., 2017). Condensin, on the other hand, is crucial for re-organizing DNA into compact cylindrical chromatids specifically during mitosis (Hirano, 2006).

Two recent findings demonstrate that cohesin and condensin must operate using similar principles. First, cohesin can also organize DNA into chromatids, albeit during interphase (Klein et al., 1999, Tedeschi et al., 2013) and only when its turnover on chromatin is abrogated by inactivation of a regulatory protein called Wapl. Second, cohesin’s association with (Ciosk et al., 2000) and dissociation from (Beckouët et al., 2016) chromosomes are regulated by three related hook-shaped proteins composed of HEAT repeats, namely Pds5, Scc3/SA, and Scc2/Nipbl. All three are monophyletic with equivalent subunits in condensin. This class of regulatory subunit called HAWKs (HEAT repeat proteins associated with kleisins) distinguishes cohesin and condensin (Wells et al., 2017) from bacterial Smc/kleisin complexes and the eukaryotic Smc5/6 complex, whose kleisin subunits associate instead with tandem winged helical domain proteins called Kleisin interacting winged-helix tandem elements (KITEs) (Palecek and Gruber, 2015).

The chromodynamics of cohesin are determined by three processes: loading; translocation; and release. The mechanism of release has been well documented (Beckouët et al., 2016, Chan et al., 2012, Lee et al., 2016). However, loading is less well understood. What is clear is that loading depends on Scc2’s hook-shaped C-terminal domain and an unstructured N-terminal domain (NTD) that snakes through a smaller Scc4 subunit composed of a superhelical array of 13 tetratricopeptide repeats (TPRs) (Ciosk et al., 2000, Hinshaw et al., 2015, Hu et al., 2015). Because Scc3 is required for cohesin’s stable association with chromosomes, it might also be involved in the loading process. In contrast, neither Pds5 (Chan et al., 2013) nor Wapl, which bind each other, are necessary. Crucially, loading requires engagement of Smc1’s and Smc3’s ATPase heads in the presence of ATP as well as the latter’s subsequent hydrolysis (Hu et al., 2011). Translocation of complexes that have just loaded could be driven either by an extrinsic force, such as RNA polymerase (Busslinger et al., 2017, Ocampo-Hafalla et al., 2016), or by an intrinsic motor associated with cohesin’s ATPase, as recently observed for condensin *in vitro* (Terakawa et al., 2017).

In *S. cerevisiae*, there are broadly two populations of chromosomal cohesin complexes: those loaded throughout chromosomes (arm cohesin) and those loaded under the control of their 120-bp point centromeres (*CEN*s), which are responsible for loading the bulk of cohesin that accumulates in peri-centric sequences 30 kb either side of each centromere (Fernius and Marston, 2009, Hu et al., 2011, Weber et al., 2004). Cohesin appears to translocate into peri-centric sequences soon after loading at *CEN*s, and as a consequence, few if any of its subunits accumulate to high levels at *CEN*s themselves. In contrast, Scc2, which is not currently considered a bona fide cohesin subunit but merely a factor required for loading, is concentrated solely at *CEN*s (Hu et al., 2015), presumably because these are sites at which loading takes place at especially high rates. Whether Scc2 accumulates at *CEN*s as a component of cohesin complexes undergoing loading or is merely targeted to *CEN*s through association of its Scc4 subunit with inner kinetochore proteins is not known. Remarkably, cohesin complexes containing versions of Smc1 (Smc1E1158Q) or Smc3 (Smc3E1155Q) that can bind, but not hydrolyze, ATP also associate preferentially at *CEN*s (Hu et al., 2015). Live-cell imaging shows that, like Scc2, they do so only in a transient manner (Hu et al., 2011). It has therefore been suggested that engagement of cohesin’s ATPase heads in the presence of ATP permits, or indeed actually triggers, cohesin’s association with *CEN* loading sites along with Scc2 but that hydrolysis is required to complete the reaction in a manner that permits translocation into neighboring chromatin.

The experiments described here suggest that cohesin switches between two states: one with Pds5 bound to Scc1 with little or no ATPase activity and a second with greatly elevated ATPase activity due to Pds5’s replacement by Scc2. The importance of this process during loading and translocation is supported by the behavior of Scc1 and Scc2 mutants that alter the way these two proteins interact. We suggest that Scc2 should no longer be considered merely as a loading factor but as a bona fide cohesin subunit whose replacement of its fellow HAWK Pds5 promotes loading, and possibly also translocation, through stimulating cohesin’s ATPase activity. Crucially, we demonstrate that, among HAWKs, Scc2 alone is both necessary and sufficient for stimulating cohesin’s DNA-dependent ATPase activity.

## Results

### Scc2 Is Necessary and Sufficient to Stimulate DNA-Dependent ATPase Activity Associated with Cohesin’s Trimeric Ring

**Figure 1:**
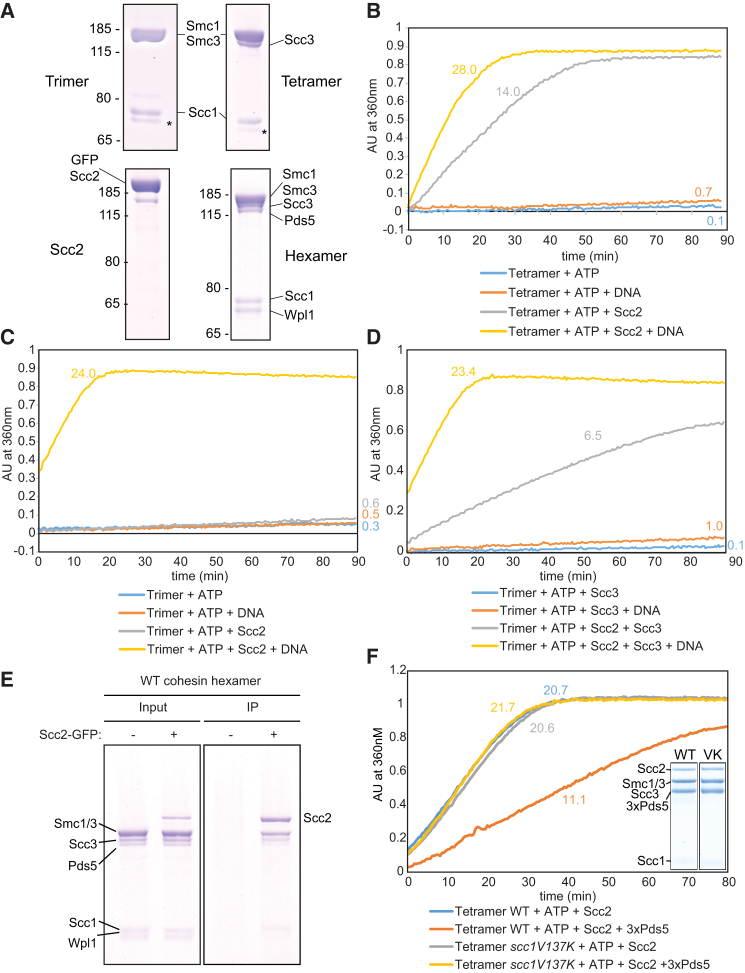
Scc2 Drives Cohesin’s DNA-Dependent ATPase (A) Cohesin trimers (Smc1, Smc3, and Scc1), tetramers (Smc1, Smc3, Scc1, and Scc3), hexamers (Smc1, Smc3, Scc1, Scc3, Pds5, and Wapl), and GFP-Scc2 were affinity purified from Sf9 cell cultures using Strep-trap columns followed by gel filtration. ^∗^ denotes a small amount of degradation of Scc1. (B) Purified tetramers were incubated with DNA, Scc2, or both and the reaction initiated by adding ATP. Rates were calculated by measuring the change in absorption at 360 nm over time. (C) ATPase activity of trimers. (D) Effect of Scc3 on ATPase of trimers. (E) Coimmunoprecipitation (co-IP) of Scc2-GFP with wild-type (WT) hexamers. Input (1/10^th^ of reaction) and IP samples were analyzed by Coomassie staining following SDS-PAGE. (F) Effect of three-fold excess of Pds5 on ATPase of wild-type and *scc1V137K* tetramers.

To address the role of cohesin’s three HAWK subunits in modulating its ATPase, we purified three types of yeast cohesin rings from insect cells: trimers containing Smc1, Smc3, and Scc1; tetramers containing Smc1, Smc3, Scc1, and Scc3; and hexamers containing Smc1, Smc3, Scc1, Scc3, Pds5, and Wapl (Figures 1A and S1A). Little or no ATPase activity was associated with any of these, even in the presence of DNA (Figures 1B, 1C, and S1B). However, activity associated with tetramers and hexamers was greatly stimulated by addition of a version of Scc2 whose N-terminal Scc4-binding domain was replaced by GFP (GFP-Scc2) and increased further still by DNA (Figures 1B and S1B). Importantly, this activity was abolished by Smc3E1155Q Smc1E1158Q double mutations that bind, but not hydrolyze, ATP (Smc1/3 EQ; Figure S1C). In contrast, GFP-Scc2 barely affected activity associated with trimers (in the absence of DNA) but stimulated it upon addition of Scc3 purified from *E. coli* (Figures 1C and 1D). Thus, at least in the absence of DNA, Scc3 enhances Scc2’s ability to stimulate cohesin’s ATPase.

In contrast to Scc2, Pds5 had no effect on the ATPase activity of tetramers, with or without DNA (Figure S1D). Remarkably, in the presence of DNA, GFP-Scc2 stimulated ATPase activity associated with trimers to a level comparable to that of tetramers and hexamers treated with GFP-Scc2 (Figure 1C). These observations imply that, among cohesin’s HAWKs, Scc2 is not only necessary for cohesin’s ATPase but also sufficient to confer its responsiveness to DNA. Scc3 clearly enhances ATPase activity, but unlike Scc2, this effect is bypassed by DNA addition. The lack of ATPase activity upon addition of Pds5 and Wapl is striking because it has been suggested that these proteins stimulate loading of *S. pombe* cohesin *in vitro* in the absence of Scc2 (Murayama and Uhlmann, 2015). Because loading *in vivo* is independent of Pds5 (see also Figure 6D) but dependent on Scc2 and abolished by Smc1/3 EQ mutations that abolish ATPase activity, we suggest that Pds5-induced loading may be an *in vitro* artifact.

Our finding that Scc2 stimulates the ATPase activity of hexamers almost as much as tetramers implies that Scc2 can associate with cohesin and stimulate its ATPase even when the complex was initially occupied by Pds5. In fact, SDS-PAGE revealed that Pds5 is selectively depleted from cohesin associated with GFP-Scc2 following the latter’s addition to wild-type or EQ hexamers in the presence of ATP (Figures 1E and S1E), under conditions where the hexamer is a stable complex (Figure S1F). Given that Pds5 and Scc2 may compete for occupancy of cohesin, we measured the effect of adding a three-fold molar excess of Pds5 to cohesin tetramers. This reduced Scc2-stimulated ATPase activity 2-fold, albeit only in the absence of DNA (Figure 1F). This inhibition was clearly due to Pds5 binding to cohesin’s kleisin subunit and not an artifact of merely adding additional protein, because tetramers that cannot bind Pds5 (Scc1V137K) were refractory to inhibition by Pds5 (Figure 1F).

### Cohesin Associates with Scc2 at *CEN*s and then Translocates into Peri-centric Sequences

The observation that Scc2 interacts with cohesin tetramers or hexamers *in vitro* raises the question as to whether these interactions occur *in vivo* and, if so, when. Strangely, chromatin immunoprecipitation sequencing (ChIP-seq) revealed that cohesin tetramers co-localize with Pds5, but not with Scc2, throughout the genome (Hu et al., 2011). The only loci where Scc2 can be reliably detected using calibrated ChIP-seq is at *CEN*s (Hu et al., 2015), which are known to confer loading of most peri-centric cohesin. This suggests that Scc2 might only be fleetingly present during the act of loading. A key question is whether Scc2 associated with *CEN* loading sites is recruited there by the Ctf19 complex (Fernius and Marston, 2009) independently of cohesin or whether Scc2 is instead bound transiently to cohesin rings undergoing loading reactions at *CEN*s.

**Figure 2:**
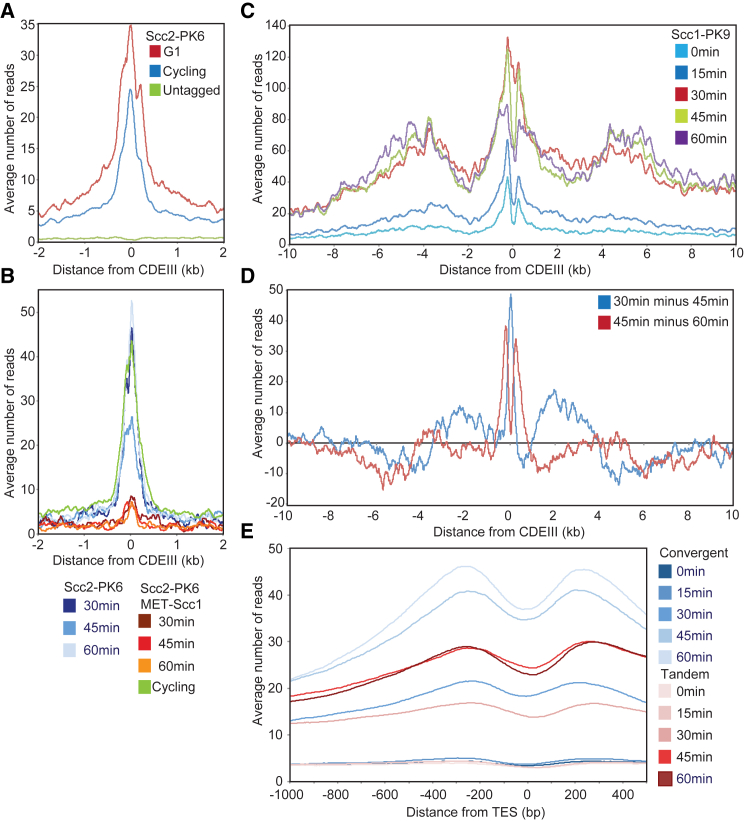
Cohesin Associates with Scc2 at *CEN*s and then Translocates into Peri-centric Sequences (A) Calibrated ChIP-seq comparing average profile of Scc2-PK6 around *CEN*s in G1 (α factor) and cycling cells. The number of reads at each base pair from *CDEIII* was averaged over all 16 chromosomes (K21388 and K699). (B) Average calibrated ChIP-seq profiles of Scc2-PK6 in cells expressing endogenous *SCC1* expressed from the *SCC1* or *MET3* promoter. Cells were arrested in G1 in the presence of methionine prior to release into methionine and nocodazole-containing medium. Samples were taken at 30, 45, and 60 min after release and from cycling cells grown in the absence of methionine (K25222 and K21388). (C) Average calibrated ChIP-seq profiles of Scc1-PK6 every 15 min from 0 to 60 min after release from G1. Data were reanalyzed from Hu et al. (2015) (GEO: GSE69907). (D) Difference plot detailing the difference in average centromere plots between time points 30, 45, and 60 min of Figure 2C. (E) Average calibrated ChIP-seq profiles of Scc1-PK6 around TESs of genes longer than 2 kb. Profiles of convergent and tandem genes are compared every 15 min from 0 to 60 min after release from G1. Data were reanalyzed from Hu et al. (2015) (GEO: GSE69907). See also Figure S2.

To address this, we compared the distributions of Scc2 and cohesin’s Scc1 subunit around centromeres using calibrated ChIP-seq in cycling and G1-arrested cells. This revealed that more not less Scc2 accumulates at *CEN*s in α-factor-arrested cells than in cycling cells (Figure 2A). Scc1 is expressed at only low levels in α-factor-arrested cells, as indeed are Scc2 and Pds5 (Figure S2B). Despite this, calibrated ChIP-seq reveals that a modest amount of cohesin is associated with peri-centric sequences (Figure 2C, light blue line), suggesting that loading does in fact occur during this stage of the cell cycle (Hu et al., 2015). Crucially, Scc2’s association with *CEN*s depends on cohesin because it is greatly reduced when cells undergo S phase in the absence of Scc1 (Figure 2B). The previous conclusion that Scc2 is absent from *CEN*s in pheromone-arrested cells as well as Scc1-depleted cells (Fernius et al., 2013) should therefore be revised.

The dependence of most peri-centric cohesin on *CEN*s and the inter-dependence of Scc1 and Scc2’s association with these sites suggest that most peri-centric cohesin complexes are derived from those loaded at *CEN*s in a reaction involving Scc2’s transient association with cohesin. Consistent with this notion, re-plotting previously published data (Hu et al., 2015) reveals that, in late G1, newly synthesized Scc1 associates initially in a peak centered on *CEN*s and subsequently translocates to neighboring peri-centric regions (Figure 2C). Thus, a plot of the difference between cohesin’s calibrated ChIP profile at 30 and 45 min, as well as 45 and 60 min, following release from pheromone reveals that a net movement of cohesin away from *CEN*s themselves and from broad peaks about 2 kb either side of them accompanies its accumulation further away in broad peaks 5 kb either side of *CEN*s (Figure 2D).

### Cohesin Loaded on Chromosome Arms Translocates from Gene Bodies to 3′ Ends

If Scc2’s preferential association with cohesin engaged in loading were a general phenomenon, then its ChIP-seq profile might also reveal where cohesin loads along chromosome arms. To address this, we plotted average values of Scc2 after aligning all genes around their transcription start or termination sites (TSSs or TESs, respectively). The average number of reads for RNA polymerase II (Pol II) genes, whether from cycling or pheromone-arrested cells, was much lower than cohesin and merely 3-fold above the untagged control. Chromosomal Scc2, as measured in this manner, was preferentially excluded from both TSSs and TESs but otherwise did not vary greatly throughout genes (Figures S2E and S2F). Though it was not greatly enriched on ribosomal protein genes or indeed on their promoters (Figure S2G), higher levels were detected close to the start sites of tRNA genes (Figure S2H), co-localizing with Smc3E1155Q containing cohesin complexes and adjacent to a peak of wild-type cohesin (Figure S2I). If these profiles reflect cohesin in the process of loading, which is uncertain, then loading must occur fairly uniformly within transcription units throughout the genome.

Given the inconclusive nature of these experiments, we reanalyzed calibrated Scc1 ChIP-seq profiles from cells released from a G1 arrest induced by α-factor (Figure 2E). This revealed that, soon after loading in late G1, cohesin is distributed uniformly across transcription units and only accumulates at the 3′ end of genes, especially convergent ones, as cells undergo S phase, an event accompanied by Smc3 acetylation and reduced turnover (Figure 2E). The simplest explanation for our data is that loading occurs throughout transcription units and not specifically at TSSs. Cohesin does not strictly speaking accumulate between convergent transcription units (Filipski and Mucha, 2002, Lengronne et al., 2004) but rather as a bimodal peak on either side of the TESs (Figure 2E).

### Scc2 Replaces Pds5 during Loading at *CEN*s

To address whether Scc2’s association with cohesin at *CEN* loading sites is accompanied by other changes in its composition, we analyzed mutant complexes containing either Smc3E1155Q or Smc1E1158Q that accumulate at *CEN*s because they bind but cannot hydrolyze ATP (Hu et al., 2011, Hu et al., 2015). Their behavior implies that ATP hydrolysis is not required for cohesin’s association with *CEN* loading sites but instead for a manner of association that permits translocation. Because accumulation at *CEN*s is abolished by *smc1S1130R* or *smc3S1128R* mutations (Hu et al., 2011), which prevent ATPase head engagement, it is thought that loading can be broken down into two steps. First, ATPase head engagement promotes co-localization of Scc2 and cohesin at *CEN*s, whereas second, ATP hydrolysis triggers stable association with and translocation along chromatin.

**Figure 3:**
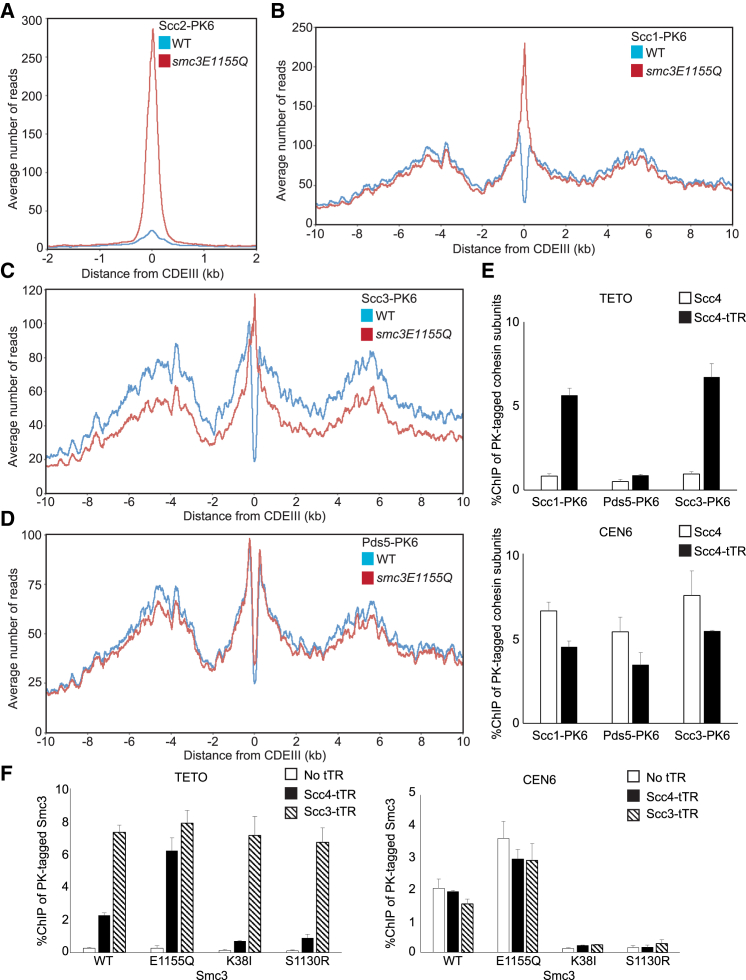
Scc2 Replaces Pds5 during Loading at *CEN*s (A–D) Average calibrated ChIP-seq profiles around *CEN*s comparing localization of (A) Scc2, (B) Scc1, (C) Scc3, and (D) Pds5 in the presence of ectopic WT or *smc3*(*E1155Q*) in cycling cells (K25467, K21388, K25370, K22005, K25373, K17438, K25376, and K19012). (E) Calibrated qPCR ChIP was used to measure association of PK-tagged Scc1, Pds5, or Scc3 with TETO on chromosome X and a sequence 400 bp from *CEN6* in Scc4-tTR/TETO cells expressing PK-tagged Scc1 (B1674), Pds5 (B1665), or Scc3 (B1625) grown to log phase. Cells with untagged Scc4 (B1627, B1635, and B1667) were used as controls. Data represent the average of three replicates, and SD is indicated. (F) The tTR-tagged Scc4 or Scc3/TETO diploid cells in which one of two *SMC3* alleles is fused with PK6 tag and mutated (B1612/B1795, B1684/B1796, B1749/B1797, and B1751/B1798) were grown at 25°C. Association of PK-tagged Smc3 mutants with TETO and centromere loci was measured by calibrated ChIP-qPCR. Cells without tTR tag (B1664, B1685, B1748, and B1750) were used as controls. Data represent the average of three replicates, and SD is indicated.

If we accept this logic and if Scc2 actually becomes part of the cohesin complex during the first step, as opposed to merely co-localizing on the chromosome, then expression of *smc3E1155Q* or *smc1E1158Q* from ectopic genes (endogenous ones are kept intact) should increase Scc2 associated with *CEN*s. Note that, in these experiments, the calibrated ChIP-seq profiles are therefore a composite of wild-type and EQ mutant cohesin. Figure 3A shows that *smc3E1155Q* expression increased Scc2’s association with *CEN*s at least ten-fold but had little effect elsewhere in the genome. It also greatly increased Scc1 at *CEN*s but had little effect elsewhere (Figure 3B). Scc3’s *CEN* recruitment was also elevated by *smc3E1155Q* (Figure 3C), albeit more modestly. In contrast, *smc3E1155Q* had little or no effect on the distribution of Pds5 (Figure 3D), implying that this particular regulatory subunit is absent from *CEN*-associated Scc1/Smc1/Smc3E1155Q/Scc2/Scc3 complexes. Similar results were obtained in cells expressing *smc1E1158Q* (Figures S3A–S3D). Scc3’s presence and Pds5’s absence from such complexes is consistent with the finding that accumulation of GFP-tagged Smc3E1155Q at centromeres depends on Scc3, but not on Pds5 (Hu et al., 2011).

To address whether Pds5 is excluded from wild-type cohesin engaged in loading, we compared the chromosomal profiles of Scc1 and Pds5 in cells arrested in late G1 when Scc1 is expressed at high levels but cohesin associated with peri-centric sequences is known to be turning over rapidly (Chan et al., 2012; Figure S3E). A scatterplot shows that Scc1 and Pds5 levels correlate highly throughout the genome. However, there is a set of sequences whose slope is half the average, namely sequences selectively depleted of Pds5, which correspond to *CEN* sequences (Figure S3F). Depletion of Pds5 from *CEN*s was less pronounced in G2/M phase, where loading is less active (Figures S3G and S3H). Because Pds5 forms a complex with cohesin when expressed in insect cells, is a constituent of soluble complexes in *Xenopus* extracts (Losada et al., 2005), and can be replaced by Scc2 *in vitro* (Figure 1E), we suggest that Scc2 displaces Pds5 upon association with cohesin at *CEN* loading sites. The ChIP-seq profiles suggest that Pds5 re-associates with cohesin and replaces Scc2 by the time the complex has translocated approximately 300 bp from the loading site.

Further evidence that Scc2’s association with cohesin *in vivo* is associated with displacement of Pds5 is our finding that tethering Scc4 to Tet operators on chromosome 14 recruits Scc1 and Scc3 but very little Pds5 to this locus (Figure 3E). Interestingly, recruitment of Smc3 to Tet operators bound by Scc4 was increased by Smc3E1155Q but greatly reduced by Smc3K38I or Smc3S1130R, implying that Scc2 interacts preferentially with cohesin, whose ATPase heads are engaged in the presence of ATP (Figure 3F). In contrast, these Smc3 mutations had little or no effect on Smc3’s recruitment to Tet operators by Scc3-TetR, suggesting that binding of Scc2 may be uniquely sensitive to the state of ATPase head engagement.

Our finding that Scc2’s association with cohesin is accompanied by loss of Pds5 and that Scc2, but not Pds5, stimulates cohesin’s ATPase suggests that it is the Scc2-bound version that is capable of loading and translocation. If so, Pds5 should be unnecessary for these processes. As predicted, Pds5 depletion had no adverse effect on either loading or translocation of cohesin, at least in late G1 cells (Figure 6D), where Pds5’s role in promoting Smc3 acetylation (Chan et al., 2013) would be immaterial. Because both Smc3E1155Q and Smc1E1158Q greatly increase the amount of Scc1 and Scc2 associated with cohesin at *CEN*s, we suggest that ATP hydrolysis is normally necessary for Scc2’s subsequent replacement by Pds5, an event that occurs to most chromosomal cohesin complexes in yeast.

In summary, the loading process at *CEN*s can be divided into two major steps. During the first, engagement of ATPase heads in the presence of ATP is accompanied by replacement of Pds5 by Scc2. During the second, ATP hydrolysis completes the reaction and leads to cohesin’s stable association with chromatin and Scc2’s replacement by Pds5. Our experiments do not address whether further rounds of ATP hydrolysis mediated by the Scc2-bound form of cohesin promote translocation into peri-centric sequences.

### Scc2 Residues Involved in DNA-Dependent ATPase Activity Are Required for the Late Loading Step

**Figure 4:**
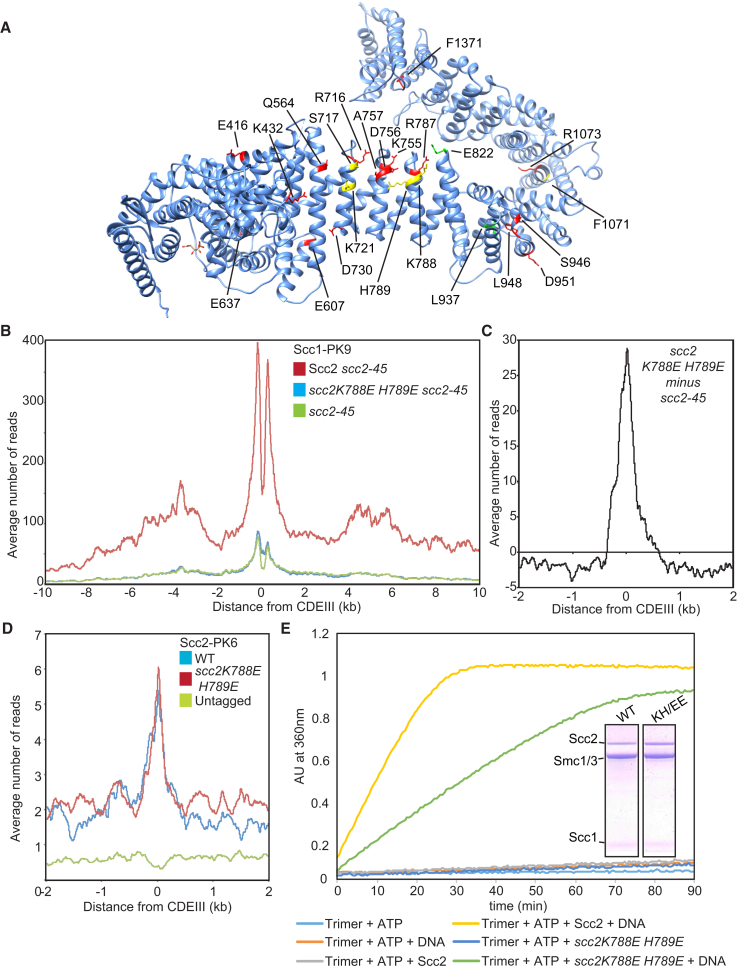
Scc2 Is Required for Both Early and Late Loading Steps (A) Residues mutated in *S. cerevisiae* Scc2 mapped onto the *C. thermophilum* structure (PDB 5T8V). Lethal mutations are in yellow, viable mutations in red, and gain-of-function mutations in green. (B) Average calibrated ChIP-seq profiles of Scc1-PK9. Cells expressing WT, *K788E H789E* double mutant, or no ectopic copy of *SCC2* over endogenous *scc2-45* were arrested in G1 at 25°C before release at 37°C into medium containing nocodazole. Samples were taken 75 min after release (K24188, K24185, and K22390). (C) The average profile of *scc2-45* was subtracted from that of *scc2E822K L937F scc2-45*, producing a difference plot revealing loading due to *scc2E822K L937F*. (D) Average calibrated ChIP-seq profiles of ectopic WT and *scc2K788E H789E* in cycling cells (K25185, K25186, and K699). (E) ATPase of WT trimers in the presence or absence of WT or mutant Scc2 and DNA. A fraction of the mix was analyzed by Coomassie staining following SDS-PAGE to confirm protein levels. See also Figure S4.

The finding that association of Smc3E1155Q with *CEN*s depends on Scc2 suggests that Scc2 is required for the first step (Hu et al., 2011). Is it also required for the second? With the aim of identifying mutations that might be preferentially defective in the second step, we created a series of mutations in highly conserved Scc2 surface residues (Figure S4A) as well as some residues mutated in Cornelia de Lange Syndrome (CdLS) patients (Table S1). Scc2 exhibits a ribbon of conservation that twists in a complex manner around the hook-shaped protein (Kikuchi et al., 2016; Figure S4A). Remarkably, no single surface amino acid change in the untagged endogenous locus was found to be lethal (Table S1), but two double mutants were, namely *S717L K721E* and *K788E H789E* (Figures 4A, 4B, and S4C). Both affect the part of Scc2 that is most conserved among HAWK subunits, the region composed of canonical HEAT repeats that, unlike the rest of the protein, are not twisted. The residues equivalent to K788 and H789 are invariably basic in a very wide variety of eukaryotes and could have a role in binding DNA.

To evaluate the effect on loading, we expressed either wild-type or *scc2K788E H789E* from an ectopic locus in cells harboring the thermosensitive (ts) *scc2-45* (*L545P D575G*) allele. Unlike wild-type, *scc2K788E H789E* failed to suppress the genome-wide loading defect of *scc2-45* cells (Figure 4B). Remarkably, it did support Scc1’s association with *CEN* loading sites (Figure 4C). Indeed, calibrated ChIP-seq revealed that the mutant protein associates with *CEN*s as efficiently as wild-type (Figure 4D), as did live imaging of GFP-tagged proteins (Figure S4D). These data suggest that Scc2K788E H789E can support the first step in the loading reaction, namely association with cohesin at *CEN*s, but cannot support stable association (loading itself) or translocation into neighboring sequences. Because wild-type is capable of both steps, it is difficult to evaluate whether the mutant is as efficient as wild-type in completing the first step.

We conclude that Scc2 is required not only for association of cohesin with engaged ATPase heads at *CEN* loading sites but also for converting these into complexes that stably associate with chromatin and are capable of translocating along it. *scc2K788E H789E* reduced by about two-fold Scc2’s ability to stimulate the ATPase activity of cohesin tetramers *in vitro*, even in the presence of DNA (Figure S4F). The defective response to DNA caused by *scc2K788E H789E* is especially apparent with trimers, which fail to respond to Scc2 unless DNA is added. Stimulation of trimer ATPase by DNA was halved by *scc2K788E H789E* (Figure 4E). Thus, K788 and H789 might have roles in the mechanism by which DNA stimulates ATPase activity associated with Scc2-bound cohesin, a process that possibly also involves binding of DNA to Scc3, Smc heads, and Smc hinges (Srinivasan et al., 2018). A crystal structure of condensin’s Ycg1 HAWK subunit associated with both DNA and the γ-kleisin Brn1 reveals that Ycg1 adopts a structure more similar to that of Scc2 than Scc3 (Kschonsak et al., 2017) and that a highly conserved R253 residue contacting phosphates on the DNA backbone corresponds to Scc2’s K788 (Figure S4B). A role in contacting DNA might therefore be a feature conserved between Scc2 and Ycg1.

### A Gain-of-Function *SCC2* Allele (*scc2E822K L937F*) that Bypasses Scc4 for Loading on Arms, but Not at *CEN*s

**Figure 5:**
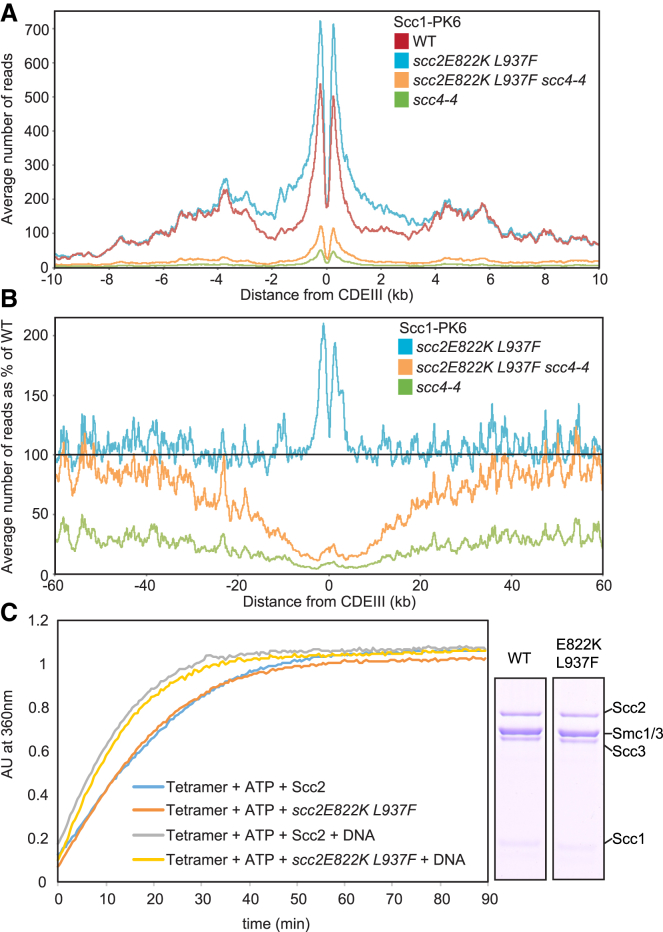
*scc2E822K L937F* Bypasses Scc4 on Arms, but Not at *CEN*s (A) Average calibrated ChIP-seq profiles of Scc1-PK6 in *SCC2 SCC4*, *scc2E822K L937F SCC4*, *SCC2 scc4-4*, and *scc2E822K L937F scc4-4* cells arrested in G1 at 25°C before release at 37°C into medium containing nocodazole. Samples were taken 75 min after release (K22005, K24687, K24744, and K22001). (B) Average calibrated ChIP profiles 60 kb either side of *CDEIII* plotted as a percentage of the average number of reads obtained for WT *SCC2 SCC4* cells in (A). (C) Effect of *scc2E822K L937F* on tetramer ATPase. A fraction of the reaction analyzed by Coomassie staining following SDS-PAGE is shown. Figure S5 shows the effect of *scc2E822K L937F* at 25°C, where its enhancement of loading is greater than at 37°C.

To explore more deeply Scc2’s role during loading and translocation, we set out to create a gain-of-function allele. Loading of cohesin at *CEN*s as well as along chromosome arms depends on Scc4 bound to Scc2’s NTD (Figure 5A). By selecting revertants of the ts allele *scc4-4* (Y40N) capable of proliferation at 37°C, we identified two *SCC2* alleles that permitted proliferation without *SCC4*. Tetrad analysis revealed that *scc2E822K* was a better suppressor than *scc2L937F* and the *scc2E822K L937F* double mutant better still (Figure S4G). E822 is a highly conserved surface residue situated on the spine of the Scc2’s most conserved HEAT repeats, very close to K788 H789 (Figures 4A, S4A, and S5A). L937 is invariably a hydrophobic residue and is buried within a HEAT repeat α helix close to the point where the protein starts to bend back on itself. Substitution by phenylalanine presumably alters the way the helix interacts with its neighbor, and bulky aromatic residues are rarely if ever found at this position. *scc2E822K L937F* enhances modestly cohesin’s association with chromosome arms as well as peri-centric sequences (Figure S5D). It also causes cohesin to accumulate to higher than normal levels in two peaks 500 bp on either side of *CEN*s, suggesting that it may retard translocation from loading sites (Figures 5B and S5D). The double mutation elevates loading along chromosome arms (defined as >30 kb from the CEN) in ts *scc4-4* mutants from ∼25% to ∼80% of wild-type when cells undergo S phase at 37°C in the presence of nocodazole but barely suppresses the loading defect at *CEN*s (Figures 5A and 5B). *scc2E822K L937F* had only a very modest if any effect on Scc2’s ability to stimulate ATPase activity associated with cohesin tetramers (Figure 5C). Thus, *scc2E822K L937F* enables cohesin to load along chromosome arms, but not at *CEN*s in the absence of Scc2’s partner Scc4.

### *SCC4* Is Essential for Loading Cohesin at *CEN*s in the Absence of Spindle Poisons, but Not in Their Presence

The failure of *scc2E822K L937F* to restore loading around centromeres in ts *scc4* mutants suggests that Scc4 may have a key role in *CEN*-specific loading, one that cannot be bypassed by *scc2E822K L937F*. This effect may be explained by the recent finding that phosphorylation of Ctf19 by DDK creates a binding site for Scc4, which presumably directs Scc2/4 to *CEN*s and promotes *CEN*-specific loading (Hinshaw et al., 2017). Surprisingly, *scc2E822K L937F* permitted substantial loading around *CEN*s in *scc4Δ* mutant cells arrested in G2/M by nocodazole (Figure S5E). Crucially, this loading also depended on *CHL4*, confirming that it is driven by *CEN* activity. Thus, in addition to Scc4-dependent loading at *CEN*s in cycling cells, a Scc4-independent mechanism promotes loading in the presence of nocodazole.

Because of these findings, we re-investigated the behavior of a *scc4* allele (*scc4*^*m35*^ F324A K327A K331A K541A K542A) thought to be defective specifically in *CEN*-specific loading. Curiously, the basis for this claim was that *scc4*^*m35*^ supposedly abolished *CEN*-specific loading in nocodazole-arrested cells (Hinshaw et al., 2015). We discovered that the residues mutated in *scc4*^*m35*^ are in fact only conserved in organisms closely related to *S. cerevisiae* and *K. lactis*, in other words in yeasts that possess point centromeres (Figure S5F). Contrary to the previous report, *scc4*^*m35*^ did not abolish *CEN*-specific loading when cells are treated with nocodazole but did so in cycling cells (Figures S5G and S5H). Our data suggest that the patch altered in *scc4*^*m35*^ may be essential for recruitment of Scc4 to *CEN*s and that this process is crucial for *CEN*-specific loading in cycling cells. Our findings suggest that recruitment of Scc4, and thereby Scc2, to *CEN*s by the Ctf19 complex co-evolved with point centromeres.

### Scc2E822K L937F Persists on Cohesin and Replaces Pds5 after Loading and Translocation

**Figure 6:**
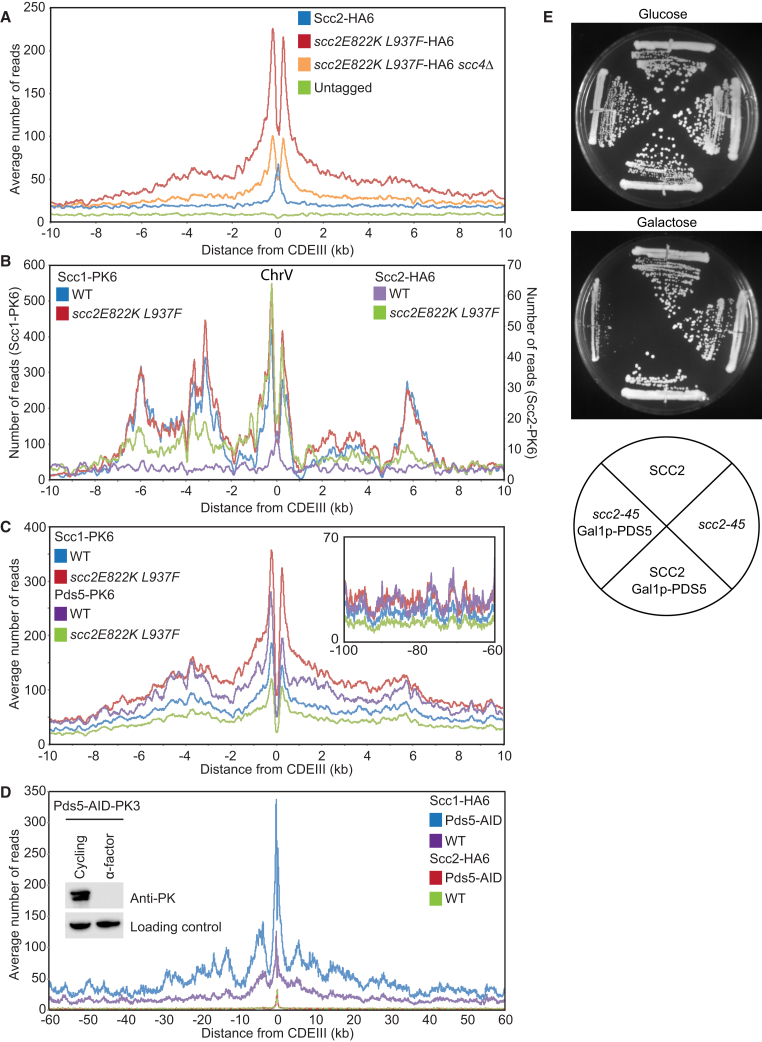
Competition between Scc2 and Pds5 Regulates Cohesin Loading Genome-wide (A) Average calibrated ChIP-seq profiles of WT Scc2-HA6 and *scc2E822K L937F*-HA6 in presence or absence of *SCC4* in cycling cells (K25054, K25053, K25418, and K699). (B) Calibrated ChIP-seq profile of ChrV comparing the localization of Scc1-PK6 and Scc2-HA6 in the presence of WT or *scc2E822K L937F* (K25054, K25053, K22005, and K24687). (C) Average calibrated ChIP-seq profiles of Scc1-PK6 or Pds5-PK6 in the presence of WT or *scc2E822K L937F* in *GAL-SIC1*-arrested cells (K26292, K26244, K25999, and K25988). (D) Average calibrated ChIP-seq profiles of Scc1-HA6 or Scc2-HA6 in the presence or absence of Pds5. Cells were arrested in late G1 using *GAL-SIC1* after addition of 5 mM auxin 30 min prior to release from pheromone. The red and green lines are identical (K26270, K26277, K26273, and K26274). (E) Pds5 overexpression hampers growth of *scc2-45* cells. WT (K699), *scc2-45* (B443), *GAL-PDS5* (B1289), and *scc2-45 GAL-PDS5* (B1282) were grown on YEP plates containing glucose or galactose at 30°C for 2 days. See Figure S5 for fluorescence-activated cell sorting (FACS) profiles.

Our data suggest that, by binding to Scc2’s NTD, Scc4 facilitates Scc2’s ability to promote loading (along chromosome arms) and that *scc2E822K L937F* alters the protein in a manner that enables now it to function without Scc4. Because *scc2E822K L937F* confers a new activity, we investigated its effect on Scc2’s association with chromosomal cohesin. Calibrated ChIP-seq revealed that *scc2E822K L937F* has a striking effect on Scc2’s distribution around *CEN*s. Instead of a narrow peak centered on *CDEIII*, the mutant protein accumulated throughout a broad peri-centric interval and especially so with a pair of symmetrical peaks on either side of *CEN*s (Figure 6A), in a manner reminiscent of cohesin itself in such cells (Figure 5A). When viewing profiles from individual chromosomes, the distribution of peri-centric Scc2E822K L937F resembled that of Scc1 in *scc2E822K L937F* cells (Figure 6B), suggesting that Scc2E822K L937F extensively co-localizes with cohesin within an interval 10 kb either side of *CEN* loading sites. No such co-localization was detected by ChIP-seq in the case of wild-type Scc2 (Figure 6B).

Importantly, *scc4Δ* greatly reduced association of Scc2E822K L937F with peri-centric sequences, which suggests that Scc2E822K L937F is bound to peri-centric cohesin, whose loading at *CEN*s was driven by Scc4’s association with Ctf19 (Figure 6A). Thus, Scc2E822K L937F associates with cohesin that has translocated from *CEN*s into peri-centric sequences. This association could arise at the time of loading and persist during translocation or it could arise *de novo* following translocation. In the latter case, one would predict that Scc2E822K L937F should co-localize with cohesin throughout the genome, which we did not observe. We therefore favor the notion that *scc2E822K L937F* enables Scc2 that had associated with cohesin at *CEN* loading to persist during its subsequent translocation.

If *scc2E822K L937F* increases occupancy of chromosomal cohesin by Scc2, and if Scc2 and Pds5 are mutually exclusive, then *scc2E822K L937F* might be expected to alter Pds5’s chromosomal distribution. This is indeed the case. Whereas *scc2E822K L937F* causes a 1.5-fold increase in the amount of Scc1 associated with peri-centric sequences, it causes a 2-fold decrease in the amount of Pds5 (Figure 6C). The net effect is that occupancy of chromosomal cohesin by Pds5 is reduced about three-fold around *CEN*s. *scc2E822K L937F* also reduced Pds5’s occupancy of cohesin along chromosome arms by about two-fold (Figure 6C). These observations demonstrate that Scc2 replaces Pds5 not only during the process of loading at *CEN*s but also during or after translocation into peri-centric sequences. Our observations suggest that Scc2E822K L937F competes with Pds5 on chromosomal cohesin more effectively than the wild-type protein.

### Pds5 Inhibits Cohesin Loading Genome-wide

Our finding that Scc2’s occupancy of chromosomal cohesin is accompanied by displacement of Pds5 suggests that the latter might act as a negative regulator of cohesin activities mediated by Scc2, namely loading and possibly also translocation. To address this, we investigated the effect of depleting Pds5 on the distributions of Scc1 and Scc2. To avoid complications associated with the fact that Pds5 is necessary for Smc3 acetylation during S phase, we analyzed the effect in cells blocked in late G1 by the Cdk1 inhibitor Sic1. Though Pds5 depletion using an Auxin-inducible degron (AID) had little or no effect on Scc2’s distribution, it had a major effect on Scc1, increasing loading throughout the genome two-fold (Figure 6D).

To address whether this effect is an indirect consequence of compromising recruitment of Wapl, whose association with Pds5 is required for cohesin turnover, we also analyzed the effect of Wapl deletion (*wpl1Δ*) at this stage of the cell cycle. Interestingly, *wpl1Δ* caused a major increase in peri-centric cohesin, an effect that is probably not due to decreased turnover, because *scc3K404E* (Beckouët et al., 2016) had little effect (Figure S6A). Importantly, both *scc3K404E* and *wpl1Δ* had little or no effect on the extent of Scc1’s association with chromosome arms whereas Pds5 depletion shows a significant increase (Figure 6D). This suggests that the increase in Scc1 association upon Pds5 depletion is due to increased loading not reduced turnover. We suggest therefore that Pds5 negatively regulates cohesin loading mediated by Scc2 throughout the genome. Consistent with this notion, overexpression of Pds5 from the *GAL* promoter causes lethality in *scc2-45* cells growing at the permissive temperature (Figure 6E). We do not understand why Pds5’s depletion does not increase Scc2’s association with the genome but suspect that, even in the absence of Pds5, Scc2’s turnover on chromosomal cohesin complexes remains too rapid for efficient formaldehyde fixation. The negative effect of Pds5 on cohesin loading genome-wide in G1 cells is consistent with our finding that Pds5 reduces Scc2’s ability to stimulate ATPase activity associated with cohesin tetramers (Figure 1F).

### Scc2 Promotes Loading, Translocation, and ATPase Activity by Interacting with Scc1

**Figure 7:**
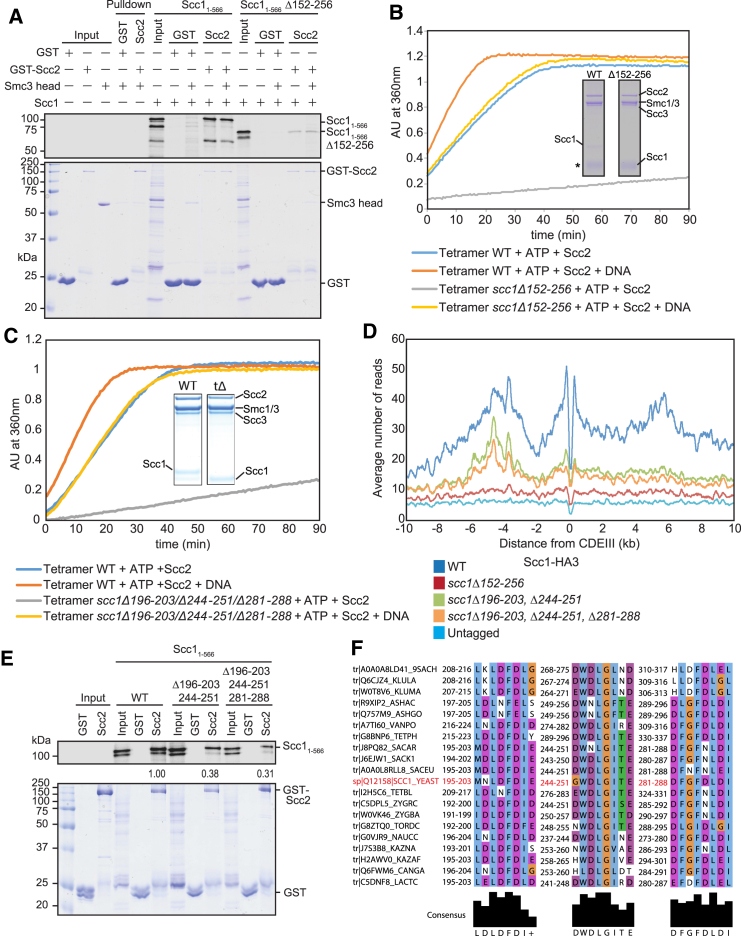
Scc2 Promotes Loading, Translocation, and ATPase Activity by Interacting with Multiple Scc1 Motifs (A) Glutathione S-transferase (GST) or GST-Scc2_171–1504_ proteins were immobilized on Glutathione Sepharose beads. Beads were incubated with ^35^S-labeled WT or Δ152–256 Scc1_1–566_ in the presence or absence of Smc3head. Input and bound proteins were separated by SDS-PAGE, stained with Coomassie (bottom), and analyzed with a phosphorimager (top). (B) Effect of *scc1Δ152–256* on tetramer ATPase activity. A fraction of the mix was then analyzed by Coomassie staining following SDS-PAGE. ^∗^The Scc1Δ152-256 protein is masked by purine nucleoside phosphorylase (PNP). (C) Effect of *scc1Δ196–203*, *Δ244–251*, and *Δ281–288* on tetramer ATPase. (D) Average calibrated ChIP-seq profiles of hemagglutinin (HA)-tagged WT and mutated Scc1 proteins in cycling cells at 25°C in the presence of untagged *SCC1* (K17184, K25896, 27148, 26992, and K699). (E) GST or GST-Scc2_171–1504_ proteins were immobilized on Glutathione Sepharose beads. Beads were incubated with ^35^S-labeled Scc1_1–566_ double mutant or Scc1_1–566_ triple mutant obtained through *in vitro* translation. Bound proteins were separated by SDS-PAGE, stained with Coomassie (bottom panel), and analyzed with a phosphorimager (top panel). The relative binding intensities of Scc1 were quantified and indicated below the autoradiograph. (F) Multiple sequence alignment showing the conservation of residues 196–203, 244–251, and 281–288 of *S. cerevisiae* Scc1 among yeasts with point centromeres. See also Figures S6 and S7.

To evaluate the importance of Scc2’s association with cohesin, we investigated its mechanism. It has been suggested on the basis of peptide arrays that Mis4 (Scc2) from *S. pombe* functions by binding to Scc3 and to the coiled coils of Smc1 and Smc3 (Murayama and Uhlmann, 2014). In contrast, Scc2 from *C. thermophilum* binds exclusively to Scc1 (residues 126–230; Kikuchi et al., 2016). The latter may be a universal mode of binding, because *S. cerevisiae* Scc2 co-precipitated with a fragment of an N-terminal fragment Scc1 containing residues 1–566 (Figure 7A). Nevertheless, there is hitherto no evidence that this Scc1:Scc2 interaction is of physiological importance, especially as many of the mutations that supposedly compromise association of *Chaetomium* Scc2 with Scc1 have no phenotype in yeast (e.g., *C.t.* Scc2 K1018E, R1053Q, and R1090T; Table S1) or, more worrying, involve substitutions to residues that are frequently found in other fungi (e.g., *C.t.* Scc2 L1373P).

Reasoning that Scc1 sequences bound by Scc2 have a role in cohesin loading, we measured the effect on yeast cell proliferation and cohesin loading of deleting Scc1 sequences other than those already known to bind Pds5 (*S.c*. Scc1 131–138), Scc3 (319–393), Smc3 (1–104), and Smc1 (483–566; Figure S6C). Tetrad analysis showed that, when expressed from an ectopic locus, all such deletions removing no more than 55 amino acids complemented *scc1Δ* (Figure S6B). However, larger deletions were not able to do so. Thus, whereas *scc1Δ152–206* and *Δ207–256* conferred viability, *Δ152–256* could not, despite binding Scc3 and forming trimeric rings with Smc1/3 (Figure S6D). Crucially, *Δ152–256* caused a severe loading defect throughout the genome (when measured in cycling cells expressing untagged wild-type protein; Figure S7A), reduced binding of Scc1 1–566 to GST-Scc2 *in vitro* (Figure 7A), and reduced Scc2-dependent ATP hydrolysis (Figure 7B), especially in the absence of DNA.

The loading defect associated with *Δ152–256* could be due either to a length requirement or redundancy of sequence motifs. In the case of the latter, it should be possible to identify, within the *207–256* interval, a motif whose deletion is lethal when combined with *Δ152–206*. Despite poor conservation among ascomycetes, we noticed that sequences within the interval 244–251 are conserved among yeasts with point centromeres, with a consensus DWDLGITE (Figure 7F). Indeed, the double mutant *Δ152–206 Δ244–251* was lethal and reduced loading *in vivo* (Figure S7A) as well as ATPase activity *in vitro* (Figure S7B).

Because *Δ180–256* is also lethal (Figure S6C), the 180–206 interval must likewise contain an element that is essential when sequences between 207 and 256 are deleted.

Interestingly, this interval contains a motif between 196 and 203 with the consensus LDLDFD, which resembles the 244–251 sequence. Moreover, a sequence with similar properties (consensus DFGFDLDI) exists within the 281–288 interval and a related sequence (consensus LDLELDFGEDID) is conserved among ascomycetes related to *C. thermophilum*, within the 126–200 interval known to bind Scc2. *scc1Δ196–203* is viable, as are *Δ244–251* and *Δ281–288* single mutants and the *scc1Δ196–203 Δ281–288* double mutant. In contrast, the *scc1Δ196–203 Δ244–251* double mutant causes poor spore viability and the *scc1Δ196–203 Δ244–251 Δ281–288* triple mutant outright lethality (Figures S6B and S6C). *scc1Δ196–203 Δ244–251 Δ281–288* reduced Scc2-dependent ATPase activity *in vitro* (Figure 7C), diminished loading of cohesin throughout the genome *in vivo* (Figure 7D), and lowered Scc1’s binding to Scc2 *in vitro* (Figure 7E).

We therefore suggest that Scc2 stimulates cohesin’s ATPase activity and loading onto chromosomes by binding to DL motifs situated within the 196–203, 244–251, and 281–288 intervals. Despite having a more modest effect on Scc2 binding (Figure 7E) and Scc2-dependent ATPase activity (Figure S7C), *scc1Δ196–203 Δ244–251* also reduced cohesin loading *in vivo* (Figure 7D), which may explain its effect on spore viability.

The three DL motifs cannot be the sole means by which Scc2 interacts with cohesin because, in the presence of DNA, Scc2 stimulates the ATPase activity of tetramers containing *scc1Δ196–203 Δ244–251 Δ281–288* (Figure 7C) or *scc1Δ152–256* (Figure 7B). Indeed, GFP-Scc2 binds to *scc1Δ152–256* tetramers *in vitro*, albeit slightly less efficiently than wild-type (Figure S6D). Given that Pds5 and Scc2 appear to compete for binding to cohesin *in vivo*, we considered that Scc2 might also interact with the Pds5-binding motif approximately 20 residues C-terminal to Scc1’s NTD. Unlike the DL motifs, this motif is highly conserved among eukaryotes (Lee et al., 2016), suggesting that it may have multiple partners. Deletion of the motif (*Δ131–138*) is lethal, as is *scc1V137K*, but we have hitherto assumed that these effects are due merely to loss of Pds5 binding. This may be mistaken. Though *scc1V137K* causes only a modest defect in cohesin loading in cells expressing wild-type Scc1 (Figure S7D), the mutation has a more severe phenotype when cells undergo S phase in Scc1’s absence. Thus, it reduced loading along chromosome arms by two-fold and around centromeres by 3.4-fold (Figure S7E). Equally striking, the cohesin that still loads at *CEN*s fails to translocate normally into peri-centric sequences and instead accumulates in two peaks 300 bp on either side of *CEN*s (Figure S7E). Under similar conditions, namely 60 min after release from pheromone-induced G1 arrest, Pds5 depletion also causes defects in cohesin loading at *CEN*s and translocation away from them but has no effect on chromosome arms (Figure S7F). Importantly, these defects are not as pronounced as those seen in the *scc1V137K*, suggesting that *scc1V137K* has an additional defect, which could be due to an effect on Scc2 binding.

## Discussion

### Scc2 and Pds5 Are Mutually Exclusive HAWKs

We show here that Scc2 is necessary for ATPase activity associated with all types of cohesin complexes, be they trimers, tetramers, or hexamers. Because cohesin loading depends on Scc2 as well as ATP hydrolysis, stimulating cohesin’s ATPase is presumably a crucial aspect of Scc2 function. Scc2 activates cohesin’s ATPase and stimulates loading by binding to specific DL motifs within Scc1. Importantly, the behavior of a gain-of-function allele *scc2E822K L937F* suggests that Scc2 can persist on cohesin during and/or subsequent to its translocation from *CEN* loading sites. This suggests that Scc2’s function may not in fact be restricted to the loading process. It might continue to regulate ATPase activity even after initial loading. If so, Scc2 should be considered a bona fide HAWK whose function depends on binding Scc1. Scc2’s association with cohesin at *CEN*s only lasts 2–4 s (Hu et al., 2011). For this reason, the vast majority of chromosomal cohesin in yeast is associated with Pds5, not Scc2. Nevertheless, we have documented instances of Pds5’s replacement by Scc2, namely at *CEN* loading sites (especially when hydrolysis is blocked), in cohesin recruited to Scc2/4 tethered at Tet operators, and when Scc2 is added to cohesin hexamers *in vitro*. Lastly, a gain-of-function *scc2E822K L937F* allele displaces a large fraction of Pds5 from cohesin throughout the genome.

Given the reciprocal nature of cohesin’s occupancy by Scc2 and Pds5, one might predict that Pds5 hinders Scc2 binding as well as vice versa. Several lines of evidence suggest that this is indeed the case. Pds5 reduces the ATPase activity of wild-type, but not *scc1V137K*, tetramers *in vitro*, its over-production is lethal to *scc2-45* cells, and Pds5 depletion leads to higher than normal Scc2-dependent loading throughout the genome. We suggest therefore that chromosomal cohesin switches between two states: one with Scc2 bound, which is active as a DNA-dependent ATPase and capable of loading and translocating along chromatin, and another with Pds5 bound, which is largely inactive as an ATPase but is capable of dissociating from chromatin in a Wapl-dependent manner. The finding that *C. thermophilum* Scc2 orthologs compete for binding Scc1 *in vitro* (Kikuchi et al., 2016), made independently during the course of our studies, suggests that competition between Scc2 and Pds5 may be a universal feature. Although much of the evidence that Scc2 replaces Pds5 concerns loading at centromeres, there is no reason to suppose that this notion does not apply throughout the genome, as suggested by the reduced association of Pds5 with chromosomal cohesin genome-wide in cells expressing Scc2E822K L937F. Because the DL motifs essential for loading mediated by Scc2 are C-terminal to the motif known to bind Pds5, it is currently unclear why Scc2 and Pds5 recruitment is mutually exclusive. As *scc1V137K* adversely affects loading and translocation as well as Pds5 recruitment, we suspect that Scc2 might also contact the Pds5 binding motif. Moreover, it is possible that Pds5 binds, in addition, the more C-terminal sequences necessary for Scc2 binding.

The view of Scc2 and Pds5 emerging from our work is difficult to reconcile with the suggestion that Pds5 and Wapl promote loading in the absence of Scc2 in living cells (Murayama and Uhlmann, 2015). As well as being unnecessary for cohesin’s ATPase activity and loading *in vivo*, Pds5 is actually absent from complexes engaged in loading. Nevertheless, we cannot exclude the possibility that, by temporarily replacing Scc2, Pds5 may facilitate further ATPase cycles mediated by Scc2 and thereby promote cohesin’s translocation along chromosomes.

### Importance of Cohesin’s ATPase

Analysis of various *smc1/3*, *scc1*, and *scc2* mutants revealed only a rough congruence between their *in vitro* ATPase activities and their abilities to load and translocate *in vivo*. On the one hand, *smc1E1158Q smc3E1155Q* or *scc1Δ196–203 Δ244–251 Δ281–288* greatly reduce both cohesin’s ATPase and its loading throughout the genome whereas *scc2E822K L937K* allele increases ATPase activity by 30% and increases loading genome-wide, especially in the absence of Scc4. On the other hand, *scc1V137K* has a profound effect on loading at and translocation from *CEN*s but has no effect on ATPase activity whereas *scc2K788E H789E* abolishes loading but reduces *in vitro* ATPase activity by only two-fold.

Curiously, the correlation between loading *in vivo* and ATPase activity *in vitro* is less marked when the latter is measured in the presence of DNA. For example, DNA greatly ameliorates the ATPase defects caused by *scc1Δ152–256* or *scc1Δ196–203 Δ244–251 Δ281–288*. We suggest that, at high concentrations *in vitro*, DNA binds to and stabilizes an “active” conformation whose creation normally depends also on other factors. DNA could do so by binding to Smc heads and/or hinges. Understanding the various mechanisms by which cohesin binds DNA, especially how these vary during the ATPase cycle, will be crucial to understanding the process of loading and translocation *in vivo*.

### Scc4 Mediates *CEN*-Specific Loading

We show here that Scc4 bound to Scc2’s N-terminal sequences has a profound role in *CEN*-specific loading. Thus, in *scc2E822K L937F* cells capable of proliferating in the absence of *SCC4*, *CEN*-specific loading is entirely lacking and the pattern of peri-centric cohesin resembles that along chromosome arms. Recent observations suggest that Scc4 performs this function by binding directly to the Ctf19 complex (Hinshaw et al., 2017) and thereby facilitates at this location high rates of Scc2-driven ATPase activity. Bioinformatics suggest that the mechanism by which Ctf19 recruits Scc4 may have co-evolved with that of high rates of cohesin loading at point centromeres. Importantly, binding Ctf19 is not the sole function of Scc4, as the latter is also important for loading along chromosome arms.

### Scc2 and Loop Extrusion

The notion that cohesin’s loading and translocation along chromatin may be driven by cycles of ATP hydrolysis mediated by replacement of Pds5 by Scc2 has important implications for the mechanism of loop extrusion (LE) thought to be responsible for controlling enhancer-promoter interactions during mammalian development. The recent observation that Scc2/Nipbl associates transiently but continuously with chromosomal cohesin in mammalian cells suggests that its function may be to stimulate the ATP hydrolysis needed to drive the translocation along chromatin necessary for LE (Rhodes et al., 2017). Interestingly, Scc2 does not co-localize with cohesin at CTCF sites (Kagey et al., 2010), which have been postulated to block LE (Fudenberg et al., 2016, Sanborn et al., 2015). We therefore suggest that CTCF might block LE by somehow hindering replacement of Pds5 by Scc2, as discussed in Wutz et al. (2017). Consistently, depletion of Pds5 in human cells results in a decrease in the definition of TADs and a reduction in the number of loops between convergent CTCF sites (Wutz et al., 2017). Indeed, the exquisite sensitivity of key developmental switches revealed by Nipbl haplo-insufficiency being the cause of Cornelia de Lange syndrome may arise because the precise level of Scc2/Nipbl may determine the rate of ATP hydrolysis and thereby the processivity of LE.

## STAR★Methods

### Key Resources Table

**Table:** 

REAGENT or RESOURCE	SOURCE	IDENTIFIER
**Antibodies**		

Anti-V5 (Mouse)	BioRad	Cat# MCA1360
Anti-HA 3F10 (Rat)	Roche	Cat# 11867423001
Anti-PGK1 22C5D8 (Mouse)	Life Technologies	Cat# 459250
Anti-GFP	Roche	Cat# 1184460001

**Bacterial and Virus Strains**		

BL21(DE3) RIPL	Life technologies	Cat# C6000-03
DH10EmbacY	Vijayachandran et al., 2011	N/A

**Chemicals, Peptides, and Recombinant Proteins**		

Acid-washed glass beads	Sigma	Cat# G8722
Complete EDTA free protease inhibitor cocktail	Roche	Cat# 4693132001
Indole-3-acetic acid (IAA/Auxin)	Sigma	Cat# I3750-5G-A
Immobilon Western ECL	Millipore	Cat# WBLKS0500
Nocodazole	Sigma	Cat# M1404
PMSF	Sigma	Cat# 329-98-6
Proteinase K	Roche	Cat# 03115836001
RNase A	Roche	Cat# 10109169001
α-factor peptide	CRUK Peptide Synthesis Service	N/A
FuGENE HD Transfection reagent	Promega	Cat# E2311
Cre Recombinase	New England Biolabs	Cat# M0298S
NuPAGE 3-8% Tris-Acetate Protein Gels, 1.5 mm, 10-well	ThermoFisher	Cat# EA0378BOX
NuPAGE 4-12% Bis-Tris Protein Gels, 1.0 mm, 10-well	ThermoFisher	Cat# NP0321BOX
Protein G dynabeads	ThermoFisher	Cat# 10003D
Dynabeads M-280 Streptavidin	Invitrogen	Cat# 11205D
DNaseI	Roche	Cat# 04716728001
StrepTrap HP	Fisher Scientific	Cat# 11540654
Desthiobiotin	Fisher Scientific	Cat# 12753064
Superose 6 Increase 10/300 GL	VWR	Cat# 29-0915-96
HiLoad 16/60 Superdex 200	GE Healthcare	Cat# GE28-9893-35
Cellfectin II Reagent	Invitrogen	Cat# 10362100
Antibiotics Antimycotic Solution	Sigma Aldrich	Cat# A5955
Fetal Bovine Serum	Sigma Aldrich	Cat# 12303C
SF-900 III SFM	Life technologies	Cat# 12658027
Ni Sepharose 6 Fast Flow	GE Healthcare	Cat# 17-5318-02
Glutathione Sepharose 4B	GE Healthcare	Cat#17075605

**Critical Commercial Assays**		

AxyPrep Mag PCR Clean-up kit	Appleton Woods Ltd	Cat# AX402
ChIP Clean and Concentrator kit	Zymo Research	Cat# D5205
E-Gel SizeSelect II Agarose Gels, 2%	ThermoFisher	Cat# G661012
EnzChek phosphate assay kit	Invitrogen	Cat# E6646
Library Quantification Kit Ion Torrent Platforms	KAPA Biosystems	Cat# KR0407
NEBNext Fast DNA Library Prep Set for Ion Torrent kit	New England Biolabs	Cat# E6270L
TnT Quick Coupled Transcription/Translation System	Promega	Cat# L2080

**Deposited Data**		

Raw and analyzed data (GEO accession number)	This study	GSE106182

**Experimental Models: Cell Lines**		

Sf9 cells in Sf-900 II SFM	ThermoFisher	Cat# 11496015

**Experimental Models: Organisms/Strains**		

Yeast strains used in this study (*S. cerevisiae* W303/K699, *C. glabrata*)	Table S2	N/A

**Recombinant DNA**		

pACEbac1 *SMC1*	This study	N/A
pACEbac1 8His-*SMC3*	This study	N/A
pIDC *SCC1*-2xStrepII	This study	N/A
pIDC *SCC3*	This study	N/A
pIDS *PDS5*-Flag	This study	N/A
pIDS *WAPL*	This study	N/A
pACEbac1 *SMC1*-8xHis-*SMC3*	This study	N/A
pIDC *SCC1*-2xStrepII-*SCC3*	This study	N/A
pIDS *PDS5-*Flag-*WAPL*	This study	N/A
pACEbac1 *SMC1-*8xHis-*SMC3* / pIDC *SCC1-*2xStrepII (trimer)	This study	N/A
pACEbac1 *SMC1-*8xHis-*SMC3* / pIDC *SCC1-*2xStrepII-*SCC3* (tetramer)	This study	N/A
pACEbac1 *SMC1-*8xHis-*SMC3* / pIDC *SCC1-*2xStrepII-*SCC3* / pIDS *PDS5-*Flag-*WAPL* (hexamer)	This study	N/A
pACEbac1 GFP-(Δ1-132)*SCC2*-StrepII	This study	N/A
pGEX6p-1 GST-Scc2_171-1504_	This study	N/A
pFastbacHT 6xHis-Pds5	This study	N/A
pCS2 Scc1_1-566_	This study	N/A
pCS2 Scc1_1-566_Δ152-256	This study	N/A
pCS2 Scc1_1-256_	This study	N/A
pACEbac1 *SMC1-*8xHis-*SMC3* / pIDC *scc1(V137K)-*2xStrepII-*SCC3* (tetramer)	This study	N/A
pACEbac1 GFP-(Δ1-132)*scc2(K788E H789E)*-StrepII	This study	N/A
pACEbac1 GFP-(Δ1-132)*scc2(E822K L937F)*-StrepII	This study	N/A
pACEbac1 *SMC1-*8xHis-*SMC3* / pIDC *scc1(Δ152-256) -*2xStrepII-*SCC3* (tetramer)	This study	N/A
pACEbac1 *SMC1-*8xHis-*SMC3* / pIDC *scc1(Δ152-206, Δ244-251) -*2xStrepII-*SCC3* (tetramer)	This study	N/A
pACEbac1 *smc1(E1158Q)-*8xHis-*smc3(E1155Q)* / pIDC *SCC1-*2xStrepII-*SCC3* / pIDS *PDS5-*Flag-*WAPL* (hexamer)	This study	N/A
pACEbac1 *SMC1-*8xHis-*SMC3* / pIDC *scc1(Δ196-203, Δ244-251, Δ281-288)-*2xStrepII-*SCC3* (tetramer)	This study	N/A
pACEbac1 *SMC1-*8xHis-*SMC3* / pIDC *scc1(Δ196-203, Δ244-251)-*2xStrepII-*SCC3* (tetramer)	This study	N/A

**Software and Algorithms**		

Galaxy platform	Giardine et al., 2005	https://usegalaxy.org
FastQC	Galaxy tool version 1.0.0	https://usegalaxy.org
Trim sequences	Galaxy tool version 1.0.0	https://usegalaxy.org
Filter FASTQ	Galaxy tool version 1.0.0	https://usegalaxy.org
Bowtie2	Langmead and Salzberg, 2012; Galaxy tool version 0.2	https://usegalaxy.org
Bam to BigWig	Galaxy tool version 0.1.0	https://usegalaxy.org
Samtools	Li et al., 2009	http://samtools.sourceforge.net/
IGB browser	Nicol et al., 2009	http://bioviz.org/igb/
Filter SAM or BAM	Li et al., 2009; Galaxy tool version 1.1.0	https://usegalaxy.org
chr_position.py	This paper	https://github.com/naomipetela/nasmythlab-ngs
filter.py	This paper	https://github.com/naomipetela/nasmythlab-ngs
bcftools call	Li et al., 2009	N/A
MutationFinder.py	This paper	N/A
yeastmine.py	This paper	N/A

### Contact for Reagent and Resource Sharing

As Lead Contact, Kim A. Nasmyth is responsible for all reagent and resource requests. Please contact Kim A. Nasmyth at ashley.nasmyth@bioch.ox.ac.uk with requests and inquiries.

### Experimental Model and Subject Details

#### Yeast strains and growth conditions

All yeast strains were derived from W303 and grown in rich medium (YEP) supplemented with 2% glucose (YPD) at 25°C unless otherwise stated. Cultures were agitated at 200rpm (Multitron Standard, Infors HT). Strain numbers and relevant genotypes of the strains used are listed in Table S2.

To arrest the cells in G1, α-factor was added to a final concentration of 2mg/L/h, every 30min for 2.5h. Release was achieved by filtration wherein cells were captured on 1.2 μm filtration paper (Whatman GE Healthcare), washed with 1L YPD and resuspended in the appropriate fresh media. To arrest the cells in G2, nocodazole (Sigma) was added to the fresh media to a final concentration of 10 μg/mL and cells were incubated until the synchronization was achieved (> 95% large-budded cells). To inactivate temperature sensitive alleles, fresh media was pre-warmed prior to filtration (Aquatron, Infors HT).

To arrest cells in late G1 with *GAL-SIC1* arrest, cells were grown in YP supplemented with 2% Raffinose and α-factor was added to a final concentration of 2mg/L/h, every 30min for 2.5h. An hour before release Galactose was added to 2% of the final volume. Release was achieved by filtration wherein cells were captured on 1.2 μm filtration paper (Whatman GE Healthcare), resuspended into YPD, and incubated for 60min at 25°C.

To produce cells deficient of Scc1, the gene was placed under the *MET3*-repressible promoter. Liquid cultures were grown in minimal media supplemented with 2% glucose and 1% -MET dropout solution overnight, diluted to OD_600_ = 0.2 and allowed to grow to OD_600_ = 0.4. Cells were then collected by filtration as described above, resuspended in YPD supplemented with 8mM methionine and arrested in G1. Once arrested, the cells were collected by filtration, washed with YPD in the presence of 8mM methionine and released into the same media.

To produce cells deficient in Pds5 using the AID system, cells were arrested with α-factor as previously described. 30min prior to release, auxin was added to 5mM final concentration. Cells were then filtered as previously described and released into YPD medium containing 5mM auxin.

### Method Details

#### Screening for suppressors of scc4-4

Forty independent colonies of the parental strain (*smc1D588E*::*TRP1* YCplac33:*scc4-4*::*NATMX scc4Δ*::*HIS3* (K23983)) were picked and grown overnight at 25°C. Each was plated at 5 OD_600_ units per plate over 3 plates and incubated at 35.5°C until colonies appeared. Up to 3 colonies were picked from each plate and streaked for single colonies at 25°C before being retested for growth at 35.5°C. Those that grew at 35.5°C were checked by PCR from genomic DNA preparations for revertants of Scc4. Isolated suppressors that did not show revertant mutations were checked for 2:2 segregation and grouped into complementation groups prior to deep sequencing. To check if for the ability to rescue the deletion of Scc1, suppressors were streaked onto 5-FOA containing medium and allowed to grow for 2 days.

#### Protein gel electrophoresis and western blotting

The samples were mixed with 4X LDS sample buffer (NuPAGE Life Technologies), loaded onto 3%–8% Tris-acetate gels (NuPAGE Life Technologies) and the proteins separated using an appropriate current. The proteins were then transferred onto 0.2 μm nitrocellulose using Trans-blot Turbo transfer packs for the Trans-blot Turbo system (Bio-Rad).

**Table:** 

Primary antibody	Immunogen	Origin	Dilution
V5 (Bio-Rad)	PK	Mouse	1:1,000
3F10 (Roche)	HA	Rat	1:5,000
PGK1	PGK1	Mouse	1:5,000

For visualization the membrane was incubated with Immobilon Western Chemiluminescent HRP substrate (Millipore) before detection using an ODYSSEY Fc Imaging System (LI-COR).

#### Multiple sequence alignment

Multiple sequence alignments were created using Clustal Omega (Sievers et al., 2011).

#### Calibrated ChIP-sequencing

Cells were grown exponentially to OD_600_ = 0.5 and the required cell cycle stage where necessary. 15 OD_600_ units of *S. cerevisiae* cells were then mixed with 5 OD_600_ units of *C. glabrata* to a total volume of 45mL and fixed with 4mL of fixative (50mM Tris-HCl, pH 8.0; 100mM NaCl; 0.5mM EGTA; 1mM EDTA; 30% (v/v) formaldehyde) for 30min at RT with rotation. Fixation was quenched with 2mL of 2.5M glycine incubated at RT for 5min with rotation. The cells were then harvested by centrifugation at 3,500rpm for 3min and washed with ice-cold 1X PBS. The cells were then resuspended in 300 μL of ChIP lysis buffer (50mM HEPES-KOH, pH 8.0; 140mM NaCl; 1mM EDTA; 1% (v/v) Triton X-100; 0.1% (w/v) sodium deoxycholate; 1mM PMSF; 1 tablet/25mL protease inhibitor cocktail (Roche)) and an equal amount of acid-washed glass beads (425-600 μm Sigma) added before cells were lysed using a FastPrep-24 benchtop homogenizer (M.P. Biomedicals) at 4°C (3x 60 s at 6.5 m/s or until > 90% of the cells were lysed as confirmed by microscopy).

The soluble fraction was isolated by centrifugation at 2,000rpm for 3min then sonicated using a bioruptor (Diagenode) for 30min in bursts of 30 s on and 30 s off at high level in a 4°C water bath to produce sheared chromatin with a size range of 200-1,000bp. After sonication the samples were centrifuged at 13,200rpm at 4°C for 20min and the supernatant was transferred into 700 μL of ChIP lysis buffer. 30 μL of protein G Dynabeads (Invitrogen) were added and the samples were pre-cleared for 1h at 4°C. 80 μL of the supernatant was taken as the WCE and 5 μg of antibody (anti-PK (Bio-Rad) or anti-HA (Roche)) was added to the remaining supernatant which was then incubated overnight at 4°C. 50 μL of protein G Dynabeads were then added and incubated at 4°C for 2h before washing 2x with ChIP lysis buffer, 3x with high salt ChIP lysis buffer (50mM HEPES-KOH, pH 8.0; 500mM NaCl; 1mM EDTA; 1% (v/v) Triton X-100; 0.1% (w/v) sodium deoxycholate; 1mM PMSF), 2x with ChIP wash buffer (10mM Tris-HCl, pH 8.0; 0.25M LiCl; 0.5% NP-40; 0.5% sodium deoxycholate; 1mM EDTA; 1mM PMSF) and 1x with TE pH 7.5. The immunoprecipitated chromatin was then eluted by incubation in 120 μL of TES buffer (50mM Tris-HCl, pH 8.0; 10mM EDTA; 1% SDS) for 15min at 65°C and the collected supernatant termed the IP sample. The WCE extracts were mixed with 40 μL of TES3 buffer (50mM Tris-HCl, pH 8.0; 10mM EDTA; 3% SDS) and all samples were de-crosslinked by incubation at 65°C overnight. RNA was degraded by incubation with 2 μL RNase A (10mg/mL; Roche) for 1h at 37°C and protein was removed by incubation with 10 μL of proteinase K (18mg/mL; Roche) for 2h at 65°C. DNA was purified using ChIP DNA Clean and Concentrator kit (Zymo Research).

#### Extraction of yeast DNA for deep sequencing

Cultures were grown to exponential phase (OD_600_ = 0.5). 12.5 OD_600_ units were then collected and diluted to a final volume of 45mL before fixation as described in the protocol for ChIP-seq. The samples were treated as specified in the ChIP-seq protocol up to the completion of the sonication step whereby 80 μL of the samples were carried forward and treated as WCE samples.

#### Preparation of sequencing libraries

Sequencing libraries were prepared using NEBNext Fast DNA Library Prep Set for Ion Torrent Kit (New England Biolabs) to the manufacturers instructions. To summarize, 10-100ng of fragmented DNA was converted to blunt ends by end repair before ligation of the Ion Xpress Barcode Adaptors. Fragments of 300bp were then selected using E-Gel SizeSelect 2% Agarose gels (Life Technologies) and amplified with 6-8 PCR cycles. The DNA concentration was then determined by qPCR using Ion Torrent DNA standards (Kapa Biosystems) as a reference. 12-16 libraries with different barcodes could then be pooled together to a final concentration of 350pM and loaded onto the Ion PI V3 Chip (Life Technologies) using the Ion Chef (Life Technologies). Sequencing was then completed on the Ion Torrent Proton (Life Technologies), typically producing 6-10 million reads per library with an average read length of 190bp.

#### Data analysis, alignment and production of BigWigs

Unless otherwise specified, data analysis was performed on the Galaxy platform (Giardine et al., 2005). Quality of the reads was assessed using FastQC (Galaxy tool version 1.0.0) and trimmed as required using ‘trim sequences’ (Galaxy tool version 1.0.0). Generally, this involved removing the first 10 bases and any bases after the 200^th^ but trimming more or fewer bases may be required to ensure the removal of kmers and that the per-base sequence content is equal across the reads. Reads shorter than 50 bp were removed using Filter FASTQ (Galaxy tool version 1.0.0, minimum size: 50, maximum size: 0, minimum quality: 0, maximum quality: 0, maximum number of bases allowed outside of quality range: 0, paired end data: false) and the remaining reads aligned to the necessary genome(s) using Bowtie2 (Galaxy tool version 0.2) with the default (–sensitive) parameters (mate paired: single-end, write unaligned reads to separate file: true, reference genome: SacCer3 or CanGla, specify read group: false, parameter settings: full parameter list, type of alignment: end to end, preset option: sensitive, disallow gaps within *n-*positions of read: 4, trim *n*-bases from 5′ of each read: 0, number of reads to be aligned: 0, strand directions: both, log mapping time: false) (Langmead and Salzberg, 2012).

To generate alignments of reads that uniquely align to the *S. cerevisiae* genome, the reads were first aligned to the *C. glabrata* (CBS138, genolevures) genome with the unaligned reads saved as a separate file. These reads that could not be aligned to the *C. glabrata* genome were then aligned to the *S. cerevisiae* (sacCer3, SGD) genome and the resulting BAM file converted to BigWig (Galaxy tool version 0.1.0) for visualization. Similarly this process was done with the order of genomes reversed to produce alignments of reads that uniquely align to *C. glabrata*.

#### Visualization of ChIP-seq profiles

The resulting BigWigs were visualized using the IGB browser (Nicol et al., 2009). To normalize the data to show quantitative ChIP signal the track was multiplied by the samples occupancy ratio (OR) and normalized to 1 million reads using the graph multiply function.

In order to calculate the average occupancy at each base pair up to 60kb around all 16 centromeres, the BAM file that contains reads uniquely aligning to *S. cerevisiae* was separated into files for each chromosome using ‘Filter SAM or BAM’ (Galaxy tool version 1.1.0). A pileup of each chromosome was then obtained using samtools mpileup (Galaxy tool version 0.0.1) (source for reference list: locally cached, reference genome: SacCer3, genotype likelihood computation: false, advanced options: basic). These files were then amended using our own script ‘chr_position.py’ to assign all unrepresented genome positions a value of 0. Each pileup was then filtered using another in-house script ‘filter.py’ to obtain the number of reads at each base pair within up to 60kb intervals either side of the centromeric CDEIII elements of each chromosome. The number of reads covering each site as one successively moves away from these CDEIII elements could then be averaged across all 16 chromosomes and calibrated by multiplying by the samples OR and normalizing to 1 million reads.

#### Identification of mutations from whole genome sequencing

SNPs were called using command line on a local server. First a pileup was created using samtools mpileup (-v–skip-indels), then SNPs called using bcftools call (-v –c). To find mutations unique to a suppressor strain, lists of SNPs from the parental strain or backcrossed clones of the suppressor strain were compared to the list of SNPs from the suppressor strain. In the case of parental strains, mutations that were present in both were removed and in the case of backcrossed clones of the suppressor strain, mutations that were present in both were kept in order to identify the mutation that caused the suppression phenotype. This was done using ‘MutationFinder.py’ and the resulting lists further narrowed using ‘yeastmine.py’ which searches the *Saccharomyces* Genome Database (SGD) for genes that correspond to the position of each mutation so that those that lie outside of genes could be removed. From this it was possible to identify the mutation in each suppressor that gave rise to the suppressor phenotype.

#### ATPase assay

ATPase activity was measured by using the EnzChek phosphate assay kit (Invitrogen) by following the protocol as provided. Cohesin in various complexes and its subunits was added to a final concentration of 50nM (or as else stated in the main text) and carried out always under 50mM NaCl in the presence of 700nM 40bp dsDNA in those experiments testing the effect of duplex DNA. The reaction was started with addition of ATP to a final concentration of 1.3mM (final reaction volume: 150ul). After completion, a fraction of each reaction was run in SDS-PAGE and the gel stained with Coomassie brilliant blue in order to test that the complexes were intact throughout the experiment and that equal amounts were used when testing various mutants and conditions.

#### Recombinant yeast cohesin complex cloning

The Smc1, 8xHis-Smc3, Scc1 2xStrepII, Scc3, Pds5, Wpl1, Scc2 from *S.cerevisiae* were gene synthesized (Genscript, Thermo Fisher Scientific, Epoch Lifescience) to optimize codons for efficient recombinant protein expression. The Smc3 and Scc1 genes were synthesized with N-terminal His and C-terminal StrepII tags, respectively. Flag tag was added into Pds5 gene at the C-termini by PCR. The *SMC1*, 8xHis*SMC3*, *SCC1*2xStrepII, *SCC3*, *PDS5*Flag, *WAPL* genes were inserted into Multibac vectors (pACEBac1, pIDC or pIDS) resulting in the vectors of *SMC1*-pACEbac1, 8xHis*SMC3*-pACEbac1, *SCC1*2xStrepII-pIDC, *SCC3*-pIDC, *PDS5*Flag-pIDS, and *WAPL*-pIDS. Genes in the same Multibac vectors were combined together by Gibson assembly, and *SMC1*-8xHis*SMC3*-pACEbac1, *SCC1*2xStrepII-*SCC3*-pIDC, *PDS5*Flag-*WAPL*-pIDS were generated. The *SMC1*-8xHis*SMC3*-pACEbac1 vector was fused to the *SCC1*2xStrepII-pIDC, *SCC1*2xStrepII-*SCC3*-pIDC, *PDS5*Flag-*WAPL-*pIDS vectors by *in vitro* Cre recombinase reaction (New England Biolabs), and then the transfer vectors for trimer (*SMC1*-8xHis*SMC3*-pACEbac1/*SCC1*2xStrepII-pIDC), tetramer (*SMC1*-8xHis*SMC3*-pACEbac1/*SCC1*2xStrepII-*SCC3*-pIDC), hexamer (*SMC1*-8xHis*SMC3*-pACEbac1/*SCC1*2xStrepII-*SCC3*-pIDC/*PDS5*Flag-*WAPL*-pIDS) were generated. A similar approach was used for the GFP-ΔΝ132-*scc2*-1xStrepII bacmid developed. The transfer vectors for trimer, tetramer, and hexamer were transformed into DH10EmbacY cells (Vijayachandran et al., 2011). The isolated bacmid DNAs were transfected into Sf9 cells using Fugene HD reagent (Promega). The generated viruses were infected into Sf9 cells, and the cells were cultured at 27°C for 72h in Insect-XPRESS protein-free medium with L-glutamate (Lonza).

#### Protein purification of the cohesin and Scc2 complexes

All versions of the cohesin complexes purified bear a tween StrepII tag in the Scc1 kleisin. This is the same for the GFP-ΔΝ132-Scc2 construct used in this study except the later bears a single Strep-II tag. Typically 500ml of SF-9 insect cells were grown to ∼3 million/ml and infected with the appropriate baculovirus stock in a 1/100 dilution. Infection was monitored daily and cells harvested when lethality (assayed by the trypan blue test) reached no more than 70%–80%. Cell pellets were then frozen in liquid nitrogen and stored at 80°C. Upon thawing, the pellets were suspended in a final volume of ∼65-70ml with HNTG lysis buffer (final concentrations of: 25mM HEPES pH 8.0, NaCl 150mM, TCEP-HCl 1mM and Glycerol 10%) and the suspension was immediately supplemented with 2 dissolved tablets of Roche Complete Protease (EDTA-free), 75μg of RNase I and 7μl of DNaseI (Roche, of 10U/μl stock). The cells were then sonicated at 80% amplitude for 5 s/burst/35ml of suspension using a Sonics Vibra-Cell (3mm microtip). In total 5 bursts were given for every 35ml half of the 70ml suspension (the sonication was always performed in ethanolised ice). A spin at 235,000 x g (45,000rpm on a Ti45 fixed angle rotor) followed for 45 mins. The isolated cleared extract was supplemented with 2mM EDTA and was then used to load a 2x5ml StrepTrap HP (Fisher Scientific) column at 1ml/min in an ÄKTA Purifier 100. Wash with HNTG+PMSF 1mM+EDTA 2mM (HNTGPE) followed at 1ml/min to the point of ΔΑU_280nm_∼0 and protein elution ensued using HNTGPE+20mM desthiobiotin (Fisher Scientific) at 1ml/min. Peak fractions were analyzed using SDS-PAGE and were further purified in a Superose 6 Increase 10/300 (VWR) using HNTG as running buffer (free of EDTA/PMSF). The resulting peaks were again analyzed using SDS-PAGE and the concentration was determined in Nanodrop using A280. Protein was aliquoted and stocked typically in concentrations ranging from 1 to 3mg/ml.

#### Pulldown experiments using holocomplexes

10μg of mouse monoclonal anti-GFP antibody (Roche) were coupled to 50μl of of Protein G Dynabeads (Invitrogen) rotating at room temperature for 1hr in a final volume of 200μls per reaction/sample using wash buffer to top up (typically the wash buffer was: HEPES 100mM, NaCl 50mM, Tween 0.04%). The beads were washed twice with 1ml wash buffer using a magnet and finally suspended in 50μls of wash buffer.

In parallel, ATPase reactions with versions of the holocomplex and (excluding mock reactions) versions of the GFP-ΔΝ132-Scc2 protein were performed essentially as described elsewhere in Methods (omitting the coupled-enzyme reaction) at 25°C on a benchtop shaker typically for 45 mins. Of the 150μl reaction 1/10th was always kept as input material. The pre-coupled ProteinG-antibody dynabeads, or streptavidin dynabeads (Invitrogen) for the control experiment, were then added (50μls) and the reaction continued for another 45mins (with shaking at 900rpm at room temperature). The beads were then pulled using a magnet and a reciprocal to the input amount was kept as flow-through material. The rest of the flow-through was then discarded and 3x1ml washes ensued using a magnet with the wash buffer at 100mM NaCl (Fig.S6) or 150mM NaCl (Figures 1 and S1). The beads were finally transferred to a new tube and eluted with 50μls of 1X SDS buffer and analyzed by SDS-PAGE with Coomassie brilliant blue staining.

#### Cloning of the GST-Scc2 plasmids and purification of GST-Scc2 and Pds5

The cDNA encoding Sc Scc2_171-1504_ was subcloned into pGEX6p-1 that introduced an N-terminal GST tag. The GST-Scc2 plasmid was transformed into *Escherichia coli* strain BL21 (DE3). Protein expression was induced by 0.2mM isopropyl-d-1-thiogalactopyranoside (IPTG) at 20°C overnight. GST-Scc2 was then purified with Glutathione Sepharose 4B resin (GE Healthcare) and stored in the storage buffer (20mM Tris-HCl pH 7.5, 150mM NaCl, 1mM TCEP-HCl) at −80°C.

Full-length Sc Pds5 cDNA was subcloned into pFastbacHT vector that introduced an N-terminal His_6_-tag. The Sc Pds5 baculovirus was made with the Bac-to-Bac system (Invitrogen). For protein expression, SF-9 cells were infected with the Pds5 baculovirus and cultured for 50hr at 27°C. Cells were harvested, resuspended in buffer I (20mM Tris-HCl pH 7.5, 500mM NaCl and 20mM imidazole), and lysed by sonication. After centrifugation, the supernatant was incubated with Ni^2+^ Sepharose 6 Fast Flow resin (GE Healthcare). The Ni resin was washed with buffer II (20mM Tris-HCl pH 7.5, 1M NaCl, 20mM imidazole), and buffer III (20mM Tris-HCl pH7.5, 100mM NaCl, 20mM imidazole) and eluted with buffer IV (20mM Tris-HCl pH 7.5, 100mM NaCl, 150mM imidazole). The eluted His_6-_Pds5 protein was concentrated and applied onto HiLoad 16/60 Superdex 200 prep grade column (GE Healthcare) that had been equilibrated with buffer V (20mM Tris-HCl pH 7.5, 150mM NaCl, 1mM TCEP-HCl). The purified Pds5 protein was then concentrated to 5mg/ml using an Amicon Ultra-15 centrifugal filter unit (Millipore) and stored at −80°C.

#### Protein binding competition assay of *S. cerevisiae* Pds5 and Scc2

The Sc Scc1_1-566_ and Scc1_1-566_ Δ152-256 cDNAs were subcloned into the pCS2 vector. These plasmids were mixed with a TNT Quick Coupled Transcription Translation System (Promega) and incubated at 30°C for 90min in the presence of ^35^S-methionine. The ^35^S-labeled Scc1 proteins were mixed with Glutathione Sepharose 4B beads bound to 10μg GST-Scc2_171-1504_ in the absence or presence of 10μg ScSmc3head, and incubated for 1 h at 4°C in the binding buffer [20mM Tris-HCl pH 7.5, 150mM NaCl, 0.1% Triton X-100]. After incubation, the beads were washed 4 times with the binding buffer. The bound proteins were separated on SDS-PAGE gels, which were stained with Coomassie blue, dried and analyzed with a phosphorimager (GE Healthcare).

For the Pds5 competition assay, Sc Scc1_1-256_ was subcloned into the pCS2 vector and translated *in vitro* with the TNT Quick Coupled Translation System (Promega). The ^35^S-labeled Scc1_1-256_ protein was incubated with varying concentrations (1.2μM, 6.0μM) of Sc Pds5 for 2h at 4°C in 50μl of the binding buffer [20mM Tris-HCl pH 7.5, 150mM NaCl, 0.1% Triton X-100] in the presence of Smc3head. After incubation, the protein mixture and GST-Scc2 were added together to Glutathione Sepharose 4B beads. The reaction mixtures were further incubated for 1 h at 5°C. The beads were washed 4 times with the binding buffer. The bound proteins were separated on SDS-PAGE gels, which were stained with Coomassie blue, dried and analyzed with a phosphorimager (GE Healthcare).

### Quantification and Statistical Analysis

#### ATPase assay

ATPase activity was measured by recording absorption at 360nm every 30 s for 90min using a PHERAstar FS. ΔΑU at 360nm was translated to Pi release using an equation derived by a standard curve of KH_2_PO_4_ (EnzChek kit). Rates were calculated from the slope of the linear phase (first 10min). At least two independent biological experiments were performed for each experiment.

#### qPCR

Experiments were performed in triplicate, the data was averaged and the standard deviation calculated.

### Data and Software Availability

#### Scripts

All scripts written for this analysis method are available to download from https://github.com/naomipetela/nasmythlab-ngs.

#### Calibrated ChIP-seq data

The accession number for the calibrated ChIP-seq data (raw and analyzed) reported in this paper is GEO: GSE106182.

## Supplemental Information

Document S1. Figures S1–S7 and Tables S1 and S2

Document S2. Article plus Supplemental Information

## References

[bib1] Beckouët F., Srinivasan M., Roig M.B., Chan K.L., Scheinost J.C., Batty P., Hu B., Petela N., Gligoris T., Smith A.C. (2016). Releasing activity disengages cohesin’s Smc3/Scc1 interface in a process blocked by acetylation. Mol. Cell.

[bib2] Busslinger G.A., Stocsits R.R., van der Lelij P., Axelsson E., Tedeschi A., Galjart N., Peters J.M. (2017). Cohesin is positioned in mammalian genomes by transcription, CTCF and Wapl. Nature.

[bib3] Chan K.L., Roig M.B., Hu B., Beckouët F., Metson J., Nasmyth K. (2012). Cohesin’s DNA exit gate is distinct from its entrance gate and is regulated by acetylation. Cell.

[bib4] Chan K.L., Gligoris T., Upcher W., Kato Y., Shirahige K., Nasmyth K., Beckouët F. (2013). Pds5 promotes and protects cohesin acetylation. Proc. Natl. Acad. Sci. USA.

[bib5] Ciosk R., Shirayama M., Shevchenko A., Tanaka T., Toth A., Shevchenko A., Nasmyth K. (2000). Cohesin’s binding to chromosomes depends on a separate complex consisting of Scc2 and Scc4 proteins. Mol. Cell.

[bib6] Fernius J., Marston A.L. (2009). Establishment of cohesion at the pericentromere by the Ctf19 kinetochore subcomplex and the replication fork-associated factor, Csm3. PLoS Genet..

[bib7] Fernius J., Nerusheva O.O., Galander S., Alves Fde.L., Rappsilber J., Marston A.L. (2013). Cohesin-dependent association of scc2/4 with the centromere initiates pericentromeric cohesion establishment. Curr. Biol..

[bib8] Filipski J., Mucha M. (2002). Structure, function and DNA composition of Saccharomyces cerevisiae chromatin loops. Gene.

[bib9] Fudenberg G., Imakaev M., Lu C., Goloborodko A., Abdennur N., Mirny L.A. (2016). Formation of chromosomal domains by loop extrusion. Cell Rep..

[bib10] Giardine B., Riemer C., Hardison R.C., Burhans R., Elnitski L., Shah P., Zhang Y., Blankenberg D., Albert I., Taylor J. (2005). Galaxy: a platform for interactive large-scale genome analysis. Genome Res..

[bib11] Hinshaw S.M., Makrantoni V., Kerr A., Marston A.L., Harrison S.C. (2015). Structural evidence for Scc4-dependent localization of cohesin loading. eLife.

[bib12] Hinshaw S.M., Makrantoni V., Harrison S.C., Marston A.L. (2017). The kinetochore receptor for the cohesin loading complex. Cell.

[bib13] Hirano T. (2006). At the heart of the chromosome: SMC proteins in action. Nat. Rev. Mol. Cell Biol..

[bib14] Hu B., Itoh T., Mishra A., Katoh Y., Chan K.L., Upcher W., Godlee C., Roig M.B., Shirahige K., Nasmyth K. (2011). ATP hydrolysis is required for relocating cohesin from sites occupied by its Scc2/4 loading complex. Curr. Biol..

[bib15] Hu B., Petela N., Kurze A., Chan K.L., Chapard C., Nasmyth K. (2015). Biological chromodynamics: a general method for measuring protein occupancy across the genome by calibrating ChIP-seq. Nucleic Acids Res..

[bib16] Kagey M.H., Newman J.J., Bilodeau S., Zhan Y., Orlando D.A., van Berkum N.L., Ebmeier C.C., Goossens J., Rahl P.B., Levine S.S. (2010). Mediator and cohesin connect gene expression and chromatin architecture. Nature.

[bib17] Kikuchi S., Borek D.M., Otwinowski Z., Tomchick D.R., Yu H. (2016). Crystal structure of the cohesin loader Scc2 and insight into cohesinopathy. Proc. Natl. Acad. Sci. USA.

[bib18] Klein F., Mahr P., Galova M., Buonomo S.B., Michaelis C., Nairz K., Nasmyth K. (1999). A central role for cohesins in sister chromatid cohesion, formation of axial elements, and recombination during yeast meiosis. Cell.

[bib19] Kschonsak M., Merkel F., Bisht S., Metz J., Rybin V., Hassler M., Haering C.H. (2017). Structural basis for a safety-belt mechanism that anchors condensin to chromosomes. Cell.

[bib20] Langmead B., Salzberg S.L. (2012). Fast gapped-read alignment with Bowtie 2. Nat. Methods.

[bib21] Lee B.G., Roig M.B., Jansma M., Petela N., Metson J., Nasmyth K., Löwe J. (2016). Crystal structure of the cohesin gatekeeper Pds5 and in complex with kleisin Scc1. Cell Rep..

[bib22] Lengronne A., Katou Y., Mori S., Yokobayashi S., Kelly G.P., Itoh T., Watanabe Y., Shirahige K., Uhlmann F. (2004). Cohesin relocation from sites of chromosomal loading to places of convergent transcription. Nature.

[bib23] Li H., Handsaker B., Wysoker A., Fennell T., Ruan J., Homer N., Marth G., Abecasis G., Durbin R., 1000 Genome Project Data Processing Subgroup (2009). The sequence alignment/map format and SAMtools. Bioinformatics.

[bib24] Losada A., Yokochi T., Hirano T. (2005). Functional contribution of Pds5 to cohesin-mediated cohesion in human cells and Xenopus egg extracts. J. Cell Sci..

[bib25] Murayama Y., Uhlmann F. (2014). Biochemical reconstitution of topological DNA binding by the cohesin ring. Nature.

[bib26] Murayama Y., Uhlmann F. (2015). DNA entry into and exit out of the cohesin ring by an interlocking gate mechanism. Cell.

[bib27] Nasmyth K. (2001). Disseminating the genome: joining, resolving, and separating sister chromatids during mitosis and meiosis. Annu. Rev. Genet..

[bib28] Nicol J.W., Helt G.A., Blanchard S.G., Raja A., Loraine A.E. (2009). The Integrated Genome Browser: free software for distribution and exploration of genome-scale datasets. Bioinformatics.

[bib29] Ocampo-Hafalla M., Muñoz S., Samora C.P., Uhlmann F. (2016). Evidence for cohesin sliding along budding yeast chromosomes. Open Biol..

[bib30] Palecek J.J., Gruber S. (2015). Kite proteins: a superfamily of SMC/kleisin partners conserved across bacteria, archaea, and eukaryotes. Structure.

[bib31] Rao S.S.P., Huang S.-C., St Hilaire B.G., Engreitz J.M., Perez E.M., Kieffer-Kwon K.-R., Sanborn A.L., Johnstone S.E., Bascom G.D., Bochkov I.D. (2017). Cohesin loss eliminates all loop domains. Cell.

[bib32] Rhodes J., Mazza D., Nasmyth K., Uphoff S. (2017). Scc2/Nipbl hops between chromosomal cohesin rings after loading. eLife.

[bib33] Sanborn A.L., Rao S.S., Huang S.C., Durand N.C., Huntley M.H., Jewett A.I., Bochkov I.D., Chinnappan D., Cutkosky A., Li J. (2015). Chromatin extrusion explains key features of loop and domain formation in wild-type and engineered genomes. Proc. Natl. Acad. Sci. USA.

[bib34] Sievers F., Wilm A., Dineen D., Gibson T.J., Karplus K., Li W., Lopez R., McWilliam H., Remmert M., Söding J. (2011). Fast, scalable generation of high-quality protein multiple sequence alignments using Clustal Omega. Mol. Syst. Biol..

[bib35] Srinivasan M., Scheinost J.C., Petela N.J., Gligoris T.G., Wissler M., Ogushi S., Collier J.E., Voulgaris M., Kurze A., Chan K.-L. (2018). The cohesin ring uses its hinge to organize DNA using non-topological as well as topological mechanisms. Cell.

[bib36] Tedeschi A., Wutz G., Huet S., Jaritz M., Wuensche A., Schirghuber E., Davidson I.F., Tang W., Cisneros D.A., Bhaskara V. (2013). Wapl is an essential regulator of chromatin structure and chromosome segregation. Nature.

[bib37] Terakawa T., Bisht S., Eeftens J.M., Dekker C., Haering C.H., Greene E.C. (2017). The condensin complex is a mechanochemical motor that translocates along DNA. Science.

[bib38] Vijayachandran L.S., Viola C., Garzoni F., Trowitzsch S., Bieniossek C., Chaillet M., Schaffitzel C., Busso D., Romier C., Poterszman A. (2011). Robots, pipelines, polyproteins: enabling multiprotein expression in prokaryotic and eukaryotic cells. J. Struct. Biol..

[bib39] Weber S.A., Gerton J.L., Polancic J.E., DeRisi J.L., Koshland D., Megee P.C. (2004). The kinetochore is an enhancer of pericentric cohesin binding. PLoS Biol..

[bib40] Wells J.N., Gligoris T.G., Nasmyth K.A., Marsh J.A. (2017). Evolution of condensin and cohesin complexes driven by replacement of Kite by Hawk proteins. Curr. Biol..

[bib41] Wutz G., Várnai C., Nagasaka K., Cisneros D.A., Stocsits R.R., Tang W., Schoenfelder S., Jessberger G., Muhar M., Hossain M.J. (2017). Topologically associating domains and chromatin loops depend on cohesin and are regulated by CTCF, WAPL, and PDS5 proteins. EMBO J..

