# Supplementary material for: Scc2 Is a Potent Activator of Cohesin’s ATPase that Promotes Loading by Binding Scc1 without Pds5
Source: Mol Cell. 2018 Jun 21;70(6):1134–1148.e7. doi: 10.1016/j.molcel.2018.05.022 (PMC6028919; doi:10.1016/j.molcel.2018.05.022)
Supplement: Document S1. Figures S1–S7 and Tables S1 and S2 [file mmc1.pdf]

Molecular Cell, Volume *70*

## Supplemental Information

# Scc2 Is a Potent Activator of Cohesin's ATPase that Promotes Loading by Binding Scc1 without Pds5

**Naomi J. Petela, Thomas G. Gligoris, Jean Metson, Byung-Gil Lee, Menelaos Voulgaris, Bin Hu, Sotaro Kikuchi, Christophe Chapard, Wentao Chen, Eeson Rajendra, Madhusudhan Srinivisan, Hongtao Yu, Jan Löwe, and Kim A. Nasmyth**

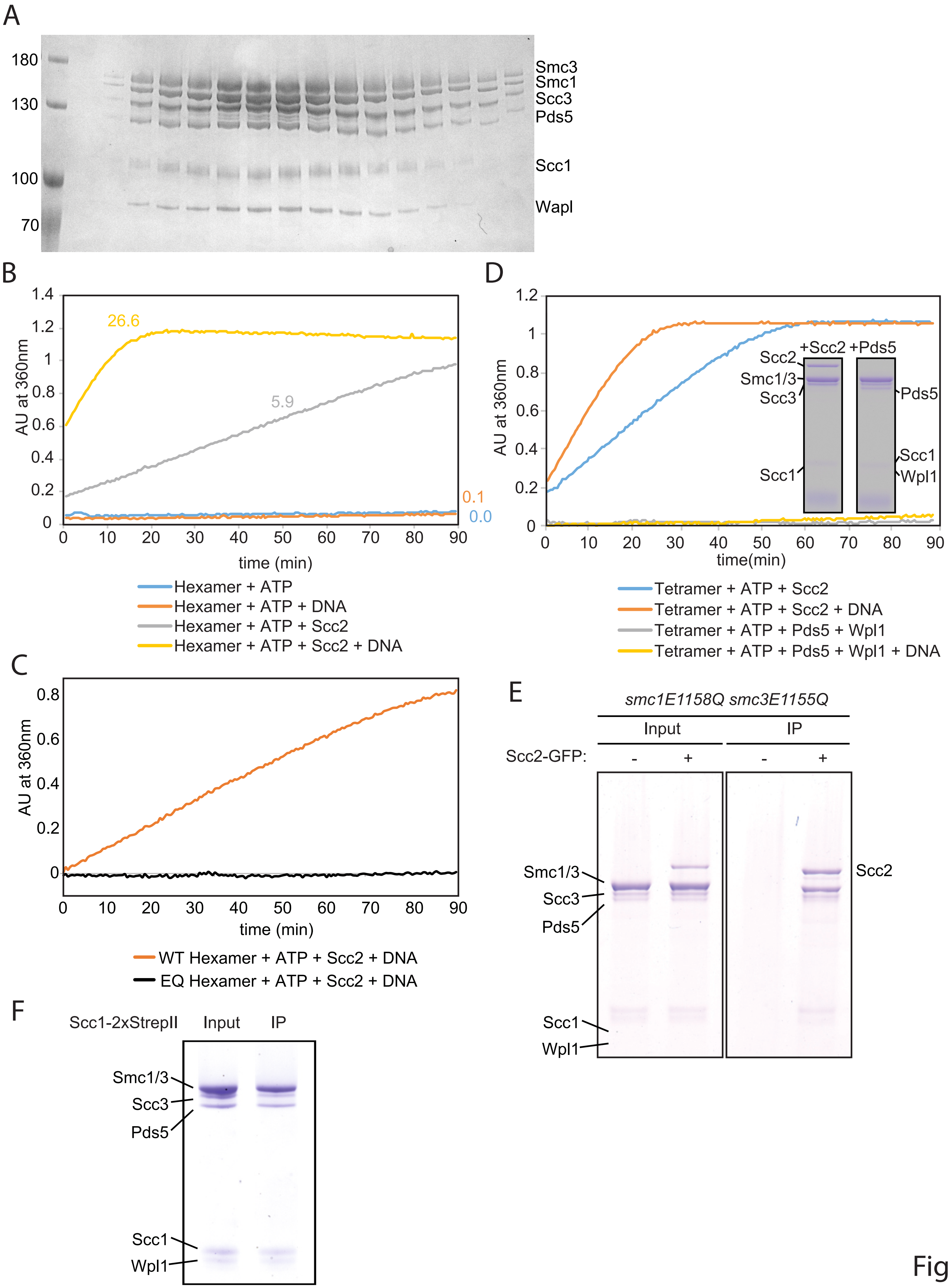
A
180
130
100
70
Smc3
Smc1
Scc3
Pds5
Scc1
Wapl
B
AU at 360nm
26.6
5.9
0.1
0.0
time (min)
Hexamer + ATP
Hexamer + ATP + DNA
Hexamer + ATP + Scc2
Hexamer + ATP + Scc2 + DNA
C
AU at 360nm
time (min)
WT Hexamer + ATP + Scc2 + DNA
EQ Hexamer + ATP + Scc2 + DNA
D
AU at 360nm
time(min)
+Scc2 +Pds5
Scc2
Smc1/3
Scc3
Scc1
Pds5
Scc1
Wpl1
Tetramer + ATP + Scc2
Tetramer + ATP + Scc2 + DNA
Tetramer + ATP + Pds5 + Wpl1
Tetramer + ATP + Pds5 + Wpl1 + DNA
E
smc1E1158Q smc3E1155Q
Input
IP
Scc2-GFP: - + - +
Smc1/3
Scc3
Pds5
Scc1
Wpl1
Scc2
F
Scc1-2xStrepII
Input
IP
Smc1/3
Scc3
Pds5
Scc1
Wpl1

Fig S1

**S1 – Related to Figure 1.**

**(A)** Purified cohesin hexamer stained with Coomassie following SDS-PAGE. **(B)** ATPase activity of hexamers. **(C)** Effect of Pds5 or Scc2 on tetramer ATPase activity in the presence or absence of DNA. **(D)** Effect of *smc3E1155Q smc1E1158Q* on hexamer ATPase activity. **(E)** Co-IP of Scc2-GFP with *smc3E1155Q smc1E1158Q* hexamers. Input (1/10$^{th}$ of reaction) and IP samples were analysed by Coomassie staining following SDS-PAGE. **(F)** Co-IP of Scc1-2xStrepII of WT hexamers performed under the same conditions as Fig. 1E and S1E. Input (1/10$^{th}$ of reaction) and IP samples were analysed by Coomassie staining following SDS-PAGE.

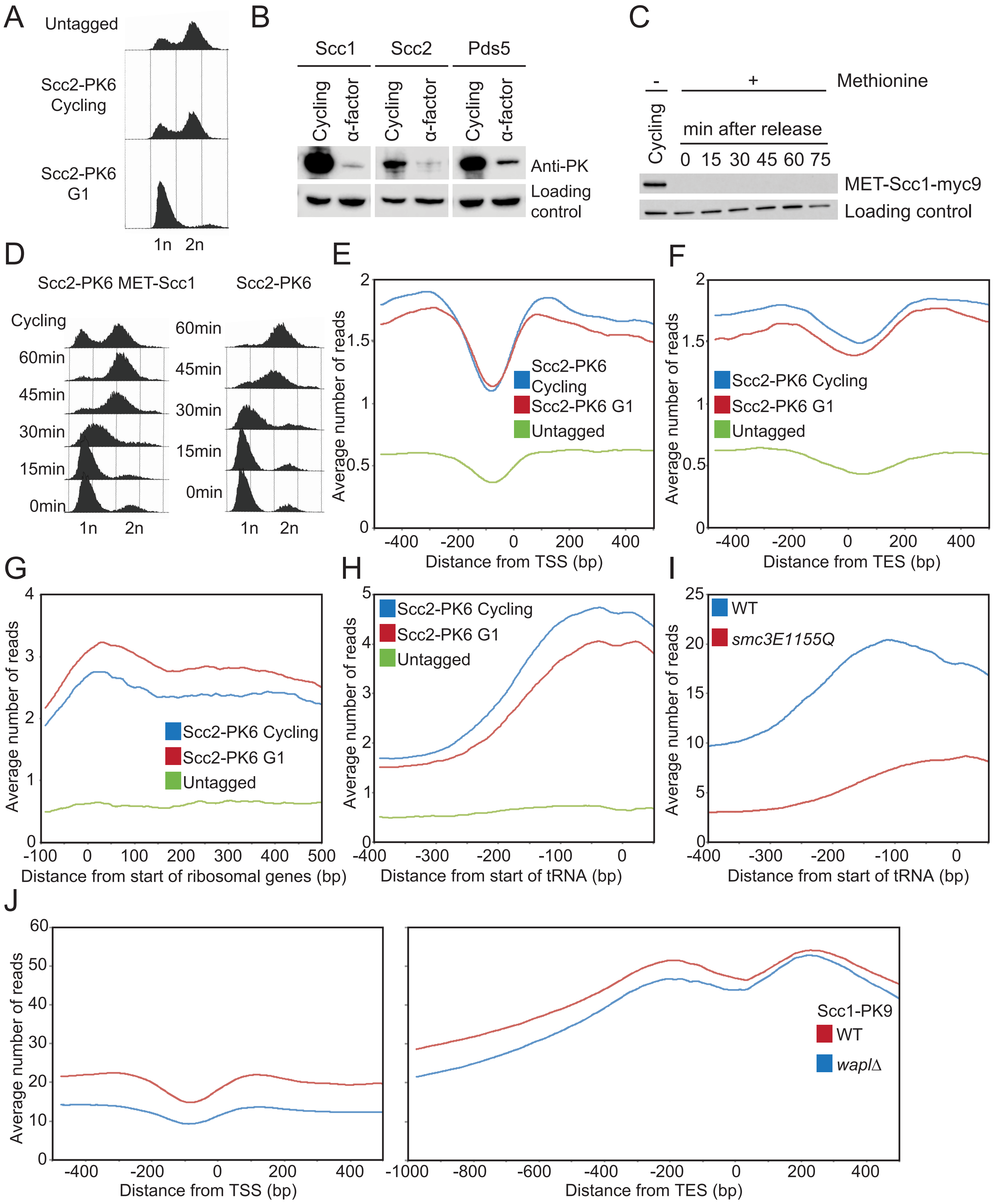

A
Untagged
Scc2-PK6 Cycling
Scc2-PK6 G1
1n 2n
B
Scc1
Scc2
Pds5
Cycling
α-factor
Anti-PK
Loading control
C
-
+
Methionine
Cycling
min after release
0 15 30 45 60 75
MET-Scc1-myc9
Loading control
D
Scc2-PK6 MET-Scc1
Scc2-PK6
Cycling
60min
45min
30min
15min
0min
1n 2n
E
Average number of reads
Scc2-PK6 Cycling
Scc2-PK6 G1
Untagged
Distance from TSS (bp)
F
Average number of reads
Scc2-PK6 Cycling
Scc2-PK6 G1
Untagged
Distance from TES (bp)
G
Average number of reads
Scc2-PK6 Cycling
Scc2-PK6 G1
Untagged
Distance from start of ribosomal genes (bp)
H
Average number of reads
Scc2-PK6 Cycling
Scc2-PK6 G1
Untagged
Distance from start of tRNA (bp)
I
Average number of reads
WT
smc3E1155Q
Distance from start of tRNA (bp)
J
Average number of reads
Distance from TSS (bp)
Distance from TES (bp)
Scc1-PK9
WT
waplΔ


Fig S2

**S2 – Related to Figure 2.**

**(A)** DNA content of Scc2-PK6 or untagged cells in G1 ($\alpha$ factor) and cycling cells (Fig 2A) was measured by FACS. (K21388, K699) **(B)** Protein levels of Scc1, Scc2 and Pds5-PK tagged cells in G1 ($\alpha$ factor) and cycling cells were assessed by Western blot. (K22005, K21388, K19012) **(C)** Western blot to assess the protein level of Scc1-myc9. Cells were arrested in G1 in the presence of methionine prior to release into methionine and nocodazole containing medium. Samples were taken every 15min after release and from cycling cells grown in the absence of methionine. The cell cycle state was measured by FACS. **(D)** (Fig 2B) (K25222). **(E-H)** Average Calibrated ChIP-seq profile of Scc2-PK6 in both cycling and G1 arrested cells around TSS **(E)** and TES **(F)** of genes longer than 2kb, start of ribosomal genes **(G)**, and start of tRNAs **(H)** (K21388). **(I)** Average calibrated ChIP-seq profile of WT or *smc3(E1155Q)*-PK6 around the start of tRNAs in cycling cells (K17407, K17409) **(J)** Average calibrated ChIP-seq profile of Scc1-PK9 in the presence or absence of Wapl around TSS and TES in *GAL-SIC1* arrested cells (K20891, K22388).

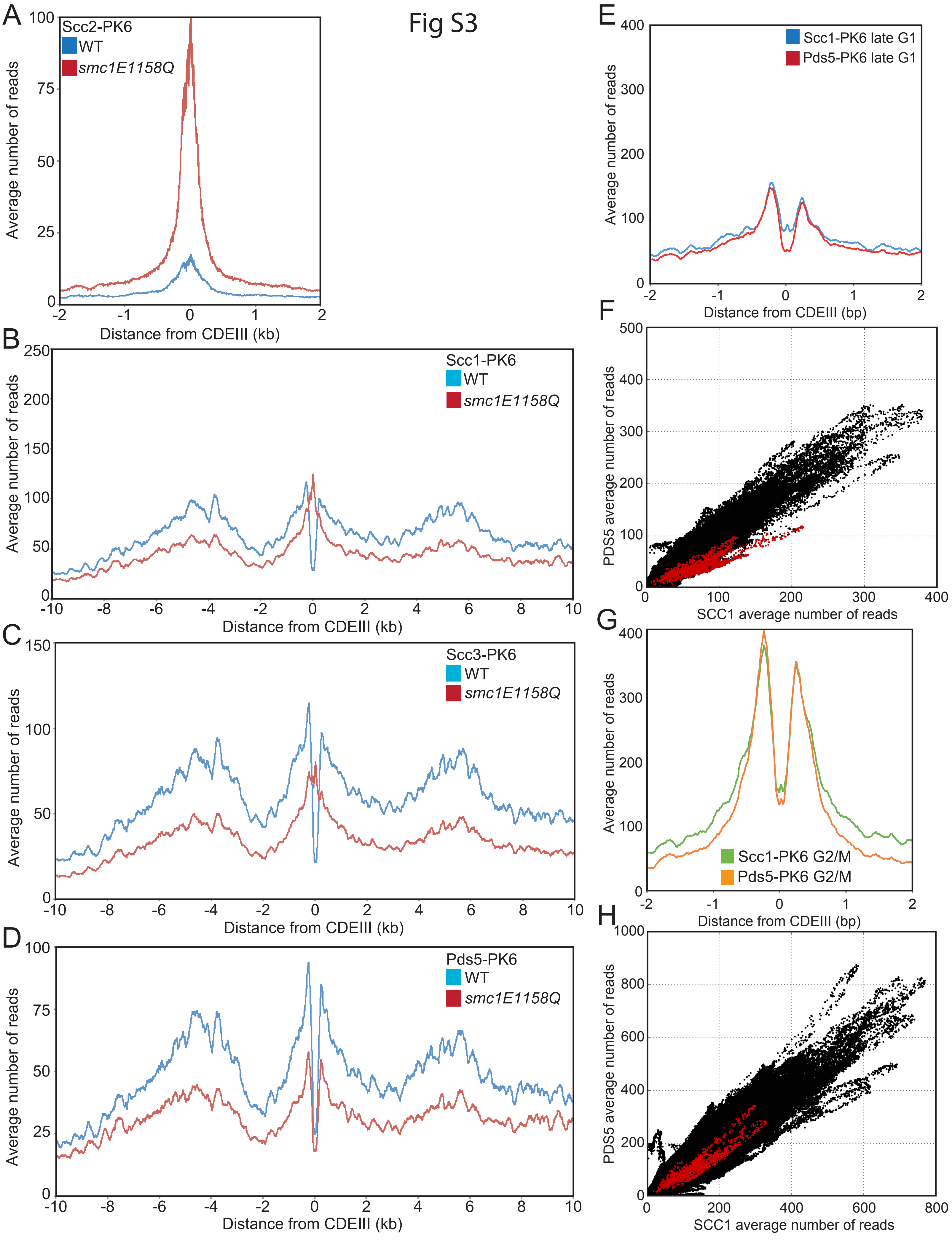

Fig S3
A
Scc2-PK6
WT
smc1E1158Q
Average number of reads
Distance from CDEIII (kb)
B
Scc1-PK6
WT
smc1E1158Q
Average number of reads
Distance from CDEIII (kb)
C
Scc3-PK6
WT
smc1E1158Q
Average number of reads
Distance from CDEIII (kb)
D
Pds5-PK6
WT
smc1E1158Q
Average number of reads
Distance from CDEIII (kb)
E
Scc1-PK6 late G1
Pds5-PK6 late G1
Average number of reads
Distance from CDEIII (bp)
F
PDS5 average number of reads
SCC1 average number of reads
G
Scc1-PK6 G2/M
Pds5-PK6 G2/M
Average number of reads
Distance from CDEIII (bp)
H
PDS5 average number of reads
SCC1 average number of reads

**S3 – Related Figure 3.**

Calibrated ChIP-seq average centromere profile comparing the localization of **(A)** Scc2, **(B)** Scc1, **(C)**, Scc3 and **(D)** Pds5 in the presence of WT or *smc1E1158Q* in cycling cells (K25644, K21388, K25640, K22005, K25637, K17438, K25652, K19012). **(E-H)** Average calibrated ChIP-seq profiles comparing Scc1 and Pds5 in **(E)** late G1 and **(G)** G2/M (K25625, K25448). Cells were arrested in late G1 with *GAL-SIC1* arrest or allowed to progress into G2 and arrested with nocodazole with samples taken 90min after release. Scatterplots comparing the average number of reads of Scc1 and Pds5 at each base across the genome in **(F)** G1 or **(H)** G2/M. Centromeric bases are coloured red.

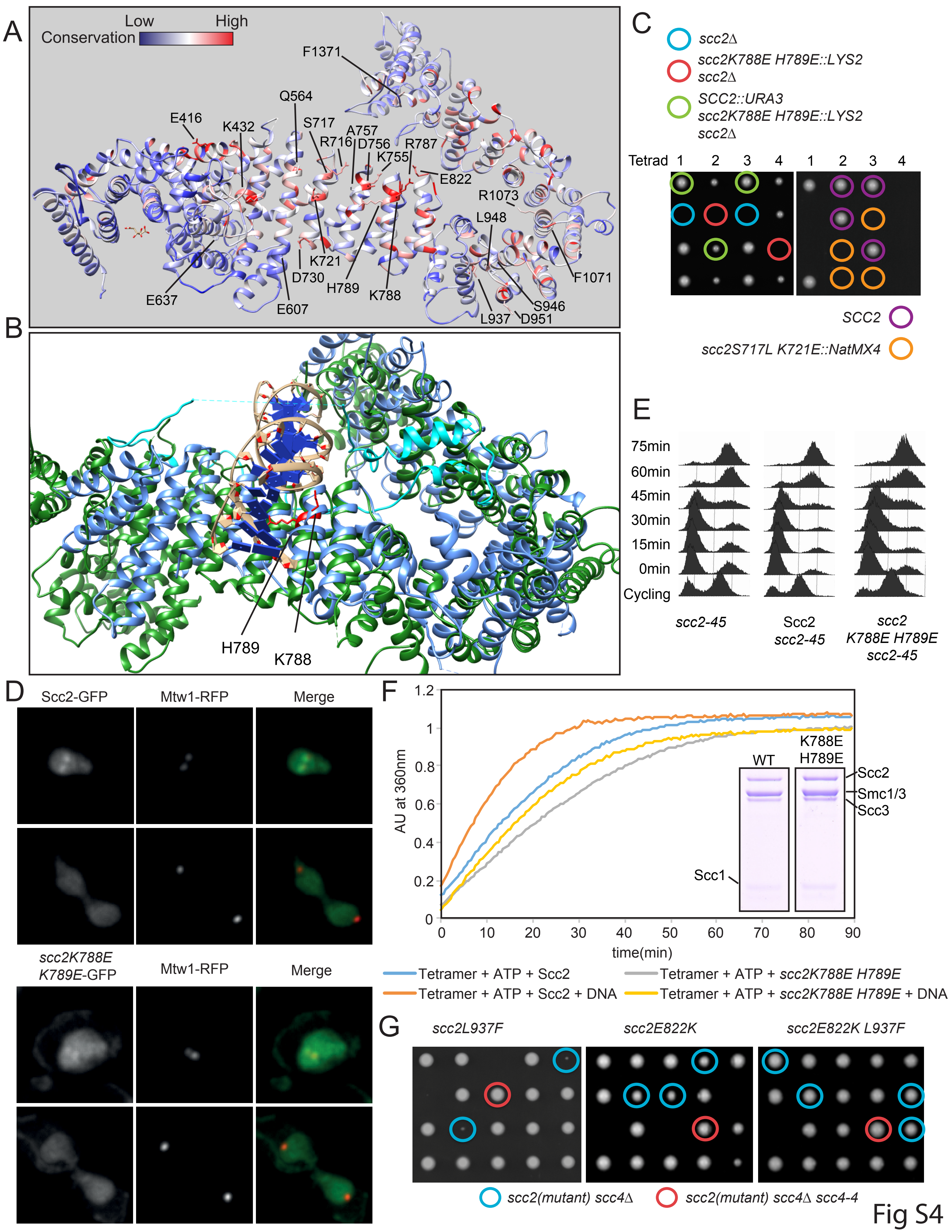
A
Low
High
Conservation
F1371
Q564
E416
K432
S717
A757
R716
D756
R787
K755
E822
R1073
L948
K721
D730
H789
K788
E637
E607
F1071
S946
L937
D951
B
H789
K788
C
scc2Δ
scc2K788E H789E::LYS2
scc2Δ
SCC2::URA3
scc2K788E H789E::LYS2
scc2Δ
Tetrad 1 2 3 4 1 2 3 4
SCC2
scc2S717L K721E::NatMX4
E
75min
60min
45min
30min
15min
0min
Cycling
scc2-45
Scc2
scc2-45
scc2
K788E H789E
scc2-45
D
Scc2-GFP
Mtw1-RFP
Merge
scc2K788E
K789E-GFP
Mtw1-RFP
Merge
F
AU at 360nm
time(min)
WT
K788E
H789E
Scc2
Smc1/3
Scc3
Scc1
Tetramer + ATP + Scc2
Tetramer + ATP + Scc2 + DNA
Tetramer + ATP + scc2K788E H789E
Tetramer + ATP + scc2K788E H789E + DNA
G
scc2L937F
scc2E822K
scc2E822K L937F
scc2(mutant) scc4Δ
scc2(mutant) scc4Δ scc4-4

Fig S4

**S4 – Related to Figure 4.**

**(A)** Crystal structure of Scc2$^{385\text{-}1840}$ of *C. thermophilum* (PDB identifier 5T8V) showing conservation of residues. Red is highly conserved and blue less conserved. Residues mutated in *S. cerevisiae* are labeled. **(B)** Crystal structure of Scc2$^{385\text{-}1840}$ (green) of *C. thermophilum* (PDB identifier 5T8V) overlaid onto *S. cerevisiae* Ycg1 (blue) (PDB identifier 5OQP). *S. cerevisiae* Scc2 residues K788 and H789 are shown in red and are predicted to contact the DNA phosphates like the equivalent residues in Ycg1. Brn1 is shown in cyan. **(C)** Tetrad dissection of diploid strains containing either *SCC2/scc2Δ lys2::scc2K788E H789E::LYS2 URA3/ura3::SCC2::URA3* or *SCC2/scc2S717L K721E::NatMX4*. Spores expressing only mutant Scc2 were not viable (circled in blue or orange respectively). **(D)** WT or *scc2K788E H789E*-GFP cells were grown to exponential phase and observed by live cell imaging. In both cases Scc2 co-localises with the kinetochore marker Mtw1-RFP. (K26872, K26875) **(E)** Cells expressing Scc1-PK9 and *scc2-45* in either the presence or absence of WT or mutant Scc2 were released from G1 ($\alpha$ factor) into nocodazole containing media at 37°C. The cell cycle state was assessed every 15mins by FACS (Fig 4B). (K24188, K24185, K22390) **(F)** ATPase activity of tetramers in the presence of WT or mutant Scc2, with and without DNA. A fraction of the reaction was analysed by Coomassie staining following SDS-PAGE to confirm protein levels. **(G)** Tetrad dissection of diploid strains containing *SCC4/scc4Δ LEU2/leu2::scc4-4::LEU2* with either *SCC2/scc2L937F, SCC2/scc2E822K* or *SCC2/scc2E822K L937F.* Spores in which *scc4Δ* is rescued by the endogenous Scc2 mutants are circled in blue.

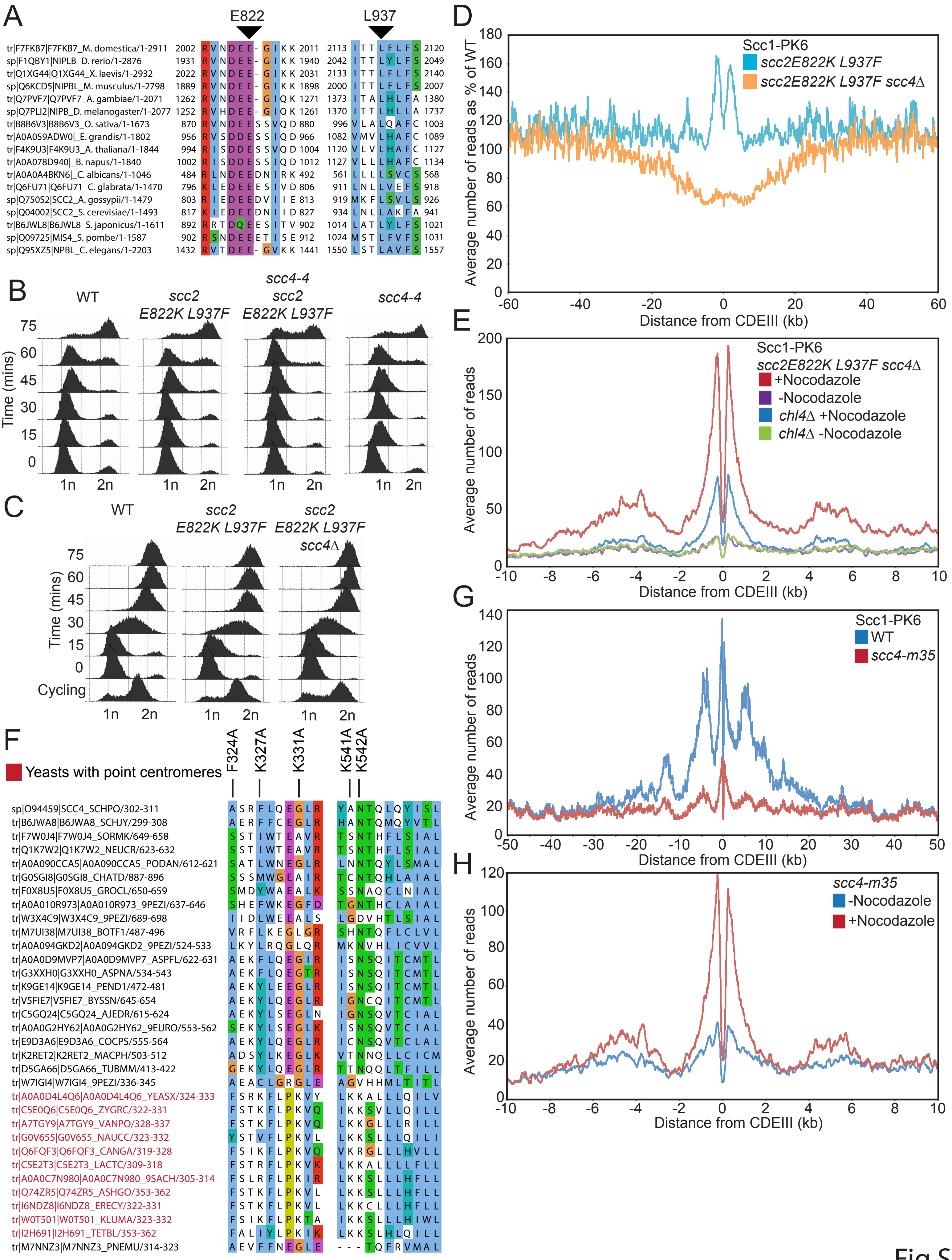
A
E822
L937
tr|F7FKB7|F7FKB7_M. domestica/1-2911
sp|F1QBY1|NIPLB_D. rerio/1-2876
tr|Q1XG44|Q1XG44_X. laevis/1-2932
sp|Q6KCD5|NIPBL_M. musculus/1-2798
tr|Q7PVF7|Q7PVF7_A. gambiae/1-2071
sp|Q7PLI2|NIPB_D. melanogaster/1-2077
tr|B8B6V3|B8B6V3_O. sativa/1-1673
tr|A0A059ADW0|_E. grandis/1-1802
tr|F4K9U3|F4K9U3_A. thaliana/1-1844
tr|A0A078D940|_B. napus/1-1840
tr|A0A0A4BKN6|_C. albicans/1-1046
tr|Q6FU71|Q6FU71_C. glabrata/1-1470
sp|Q750S2|SCC2_A. gossypii/1-1479
sp|Q04002|SCC2_S. cerevisiae/1-1493
tr|B6JWL8|B6JWL8_S. japonicus/1-1611
sp|Q09725|MIS4_S. pombe/1-1587
sp|Q95XZ5|NPBL_C. elegans/1-2203
B
WT
scc2 E822K L937F
scc4-4 scc2 E822K L937F
scc4-4
Time (mins)
1n 2n
C
WT
scc2 E822K L937F
scc2 E822K L937F scc4Δ
Time (mins)
Cycling
1n 2n
D
Scc1-PK6
scc2E822K L937F
scc2E822K L937F scc4Δ
Average number of reads as % of WT
Distance from CDEIII (kb)
E
Scc1-PK6
scc2E822K L937F scc4Δ
+Nocodazole
-Nocodazole
chl4Δ +Nocodazole
chl4Δ -Nocodazole
Average number of reads
Distance from CDEIII (kb)
F
Yeasts with point centromeres
F324A
K327A
K331A
K541A
K542A
sp|O94459|SCC4_SCHPO/302-311
tr|B6JWA8|B6JWA8_SCHJY/299-308
tr|F7W0J4|F7W0J4_SORMK/649-658
tr|Q1K7W2|Q1K7W2_NEUCR/623-632
tr|A0A090CCA5|A0A090CCA5_PODAN/612-621
tr|G0SGI8|G0SGI8_CHATD/887-896
tr|F0X8U5|F0X8U5_GROCL/650-659
tr|A0A010R973|A0A010R973_9PEZI/637-646
tr|W3X4C9|W3X4C9_9PEZI/689-698
tr|M7UI38|M7UI38_BOTF1/487-496
tr|A0A094GKD2|A0A094GKD2_9PEZI/524-533
tr|A0A0D9MVP7|A0A0D9MVP7_ASPFL/622-631
tr|G3XXH0|G3XXH0_ASPNA/534-543
tr|K9GE14|K9GE14_PEND1/472-481
tr|V5FIE7|V5FIE7_BYSSN/645-654
tr|C5GQ24|C5GQ24_AJEDR/615-624
tr|A0A0G2HY62|A0A0G2HY62_9EURO/553-562
tr|E9D3A6|E9D3A6_COCPS/555-564
tr|K2RET2|K2RET2_MACPH/503-512
tr|D5GA66|D5GA66_TUBMM/413-422
tr|W7IGI4|W7IGI4_9PEZI/336-345
tr|A0A0D4L4Q6|A0A0D4L4Q6_YEASX/324-333
tr|C5E0Q6|C5E0Q6_ZYGRC/322-331
tr|A7TGY9|A7TGY9_VANPO/328-337
tr|G0V655|G0V655_NAUCC/323-332
tr|Q6FQF3|Q6FQF3_CANGA/319-328
tr|C5E2T3|C5E2T3_LACTC/309-318
tr|A0A0C7N980|A0A0C7N980_9SACH/305-314
tr|Q74ZR5|Q74ZR5_ASHGO/353-362
tr|I6NDZ8|I6NDZ8_ERECY/322-331
tr|W0T501|W0T501_KLUMA/323-332
tr|I2H691|I2H691_TETBL/353-362
tr|M7NNZ3|M7NNZ3_PNEMU/314-323
G
Scc1-PK6
WT
scc4-m35
Average number of reads
Distance from CDEIII (kb)
H
scc4-m35
-Nocodazole
+Nocodazole
Average number of reads
Distance from CDEIII (kb)

Fig S5

**S5 – Related to Figures 5 and 6**

**(A)** Multiple sequence alignment indicating conservation of Scc2 resides E822 and L937 in *S. cerevisiae* across a wide variety of fungi. **(B)** Cells expressing Scc1-PK6 in the presence of WT or mutant Scc2 and WT or *scc4-4,* were released from G1 ($\alpha$ factor) into nocodazole containing media at 37°C. The cell cycle state was assessed every 15mins by FACS (Fig 5AB). (K22005, K24687, K24744, K22001) **(C)** Cells expressing Scc1-PK6 in the presence of WT or mutant Scc2 and in the presence or absence of Scc4, were released from G1 ($\alpha$ factor) into nocodazole containing media at 25°C. The cell cycle state was assessed every 15mins by FACS (Fig S5D). (K22005, K24687, K24745) **(D)** Average calibrated ChIP-seq profile plotted as a percentage of the average number of reads obtained for WT cells. Cells were grown as described in C and samples taken at 75min. **(E)** Average calibrated ChIP-seq profile comparing Scc1-PK6 in the presence of *scc2E822K L937F* and *scc4Δ*, with or without *chl4Δ* and nocodazole. Cycling cells were incubated with or without nocodazole for 90mins. (K25061, K24745) **(F)** Multiple sequence alignment indicating conservation of resides that are mutated in the *m35* allele of Scc4 in *S. cerevisiae.* Yeasts with point centromeres are coloured in red. **(G)** Average calibrated ChIP-seq profile comparing Scc1-PK6 in the presence or absence of *scc4-m35.* Cells were arrested in G1 ($\alpha$ factor) prior to release. Samples were taken 60min post-release. (K22005, K25049) **(H)** Average calibrated ChIP-seq profile comparing Scc1-PK6 in *scc4-m35* cells in the presence or absence of nocodazole. Cycling cells were incubated with or without nocodazole for 90mins. (K25049)

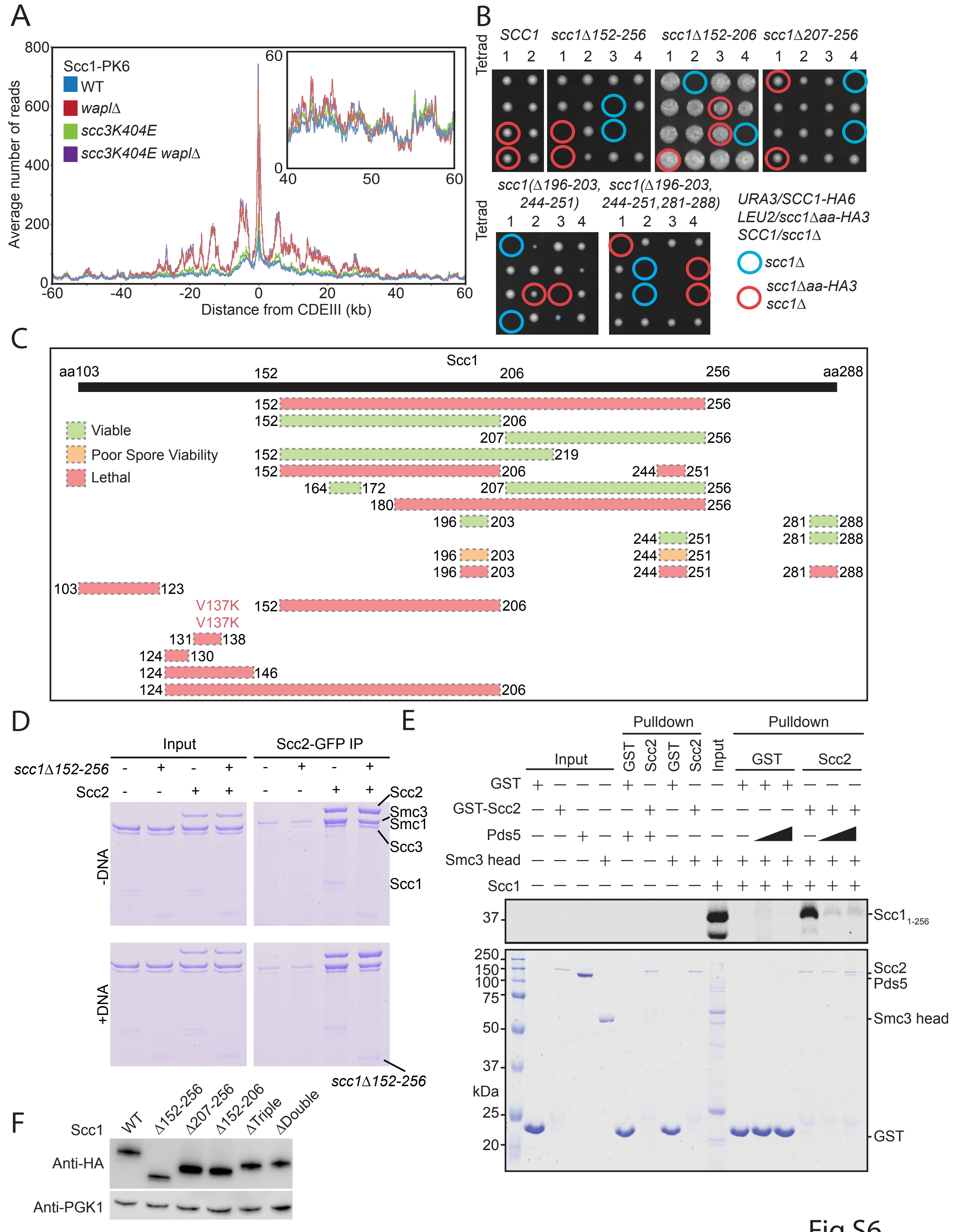
A
Scc1-PK6
WT
waplΔ
scc3K404E
scc3K404E waplΔ
Average number of reads
Distance from CDEIII (kb)
B
SCC1
scc1Δ152-256
scc1Δ152-206
scc1Δ207-256
Tetrad
scc1(Δ196-203, 244-251)
scc1(Δ196-203, 244-251,281-288)
URA3/SCC1-HA6
LEU2/scc1Δaa-HA3
SCC1/scc1Δ
scc1Δ
scc1Δaa-HA3 scc1Δ
C
Scc1
aa103
aa288
Viable
Poor Spore Viability
Lethal
V137K
D
Input
Scc2-GFP IP
scc1Δ152-256
Scc2
Smc3
Smc1
Scc3
Scc1
-DNA
+DNA
scc1Δ152-256
E
Input
Pulldown
GST
Scc2
GST-Scc2
Pds5
Smc3 head
Scc1
Scc1 1-256
Smc3 head
kDa
F
Scc1
WT
Δ152-256
Δ207-256
Δ152-206
ΔTriple
ΔDouble
Anti-HA
Anti-PGK1

Fig S6

**S6 – Related to Figure 7**

**(A)** Average calibrated ChIP-seq profile comparing Scc1-PK6 in the presence and absence of *waplΔ* and *scc3K404E* in cells arrested in late G1 with *GAL-SIC1*. (K26281, K26286, K26288, K26289). **(B)** Tetrad dissection of diploid strains containing *URA3/SCC1-HA6 LEU2/leu2::scc1Δaa-HA3::LEU2* and *SCC1/scc1Δ.* Spores in that contain *scc1Δaa* and *scc1Δ* are circled in red. **(C)** Schematic of deletions in Scc1. Lethal deletions in red and viable in green. **(D)** Co-IP of Scc2-GFP with either WT or *scc1Δ152-256* cohesin tetramers in the presence and absence of DNA. Input and IP samples were analysed by Coomassie staining following SDS-PAGE. **(E)** GST or GST-$Scc2_{171-1504}$ proteins were immobilized on Glutathione Sepharose beads. Beads were incubated with $^{35}$S-labeled $Scc1_{1-256}$ and varying concentrations of Pds5 in the presence of Smc3head. Input and bound proteins were separated by SDS-PAGE and stained with Coomassie (Bottom) and analysed with a phosphorimager (Top). **(F)** The protein levels of mutant Scc1-HA3 were compared in cycling cells by Western blot. PGK1 was used as a loading control. (Fig. 7D) (K17184, K25896, K25995, K25997, K27148, K26992).

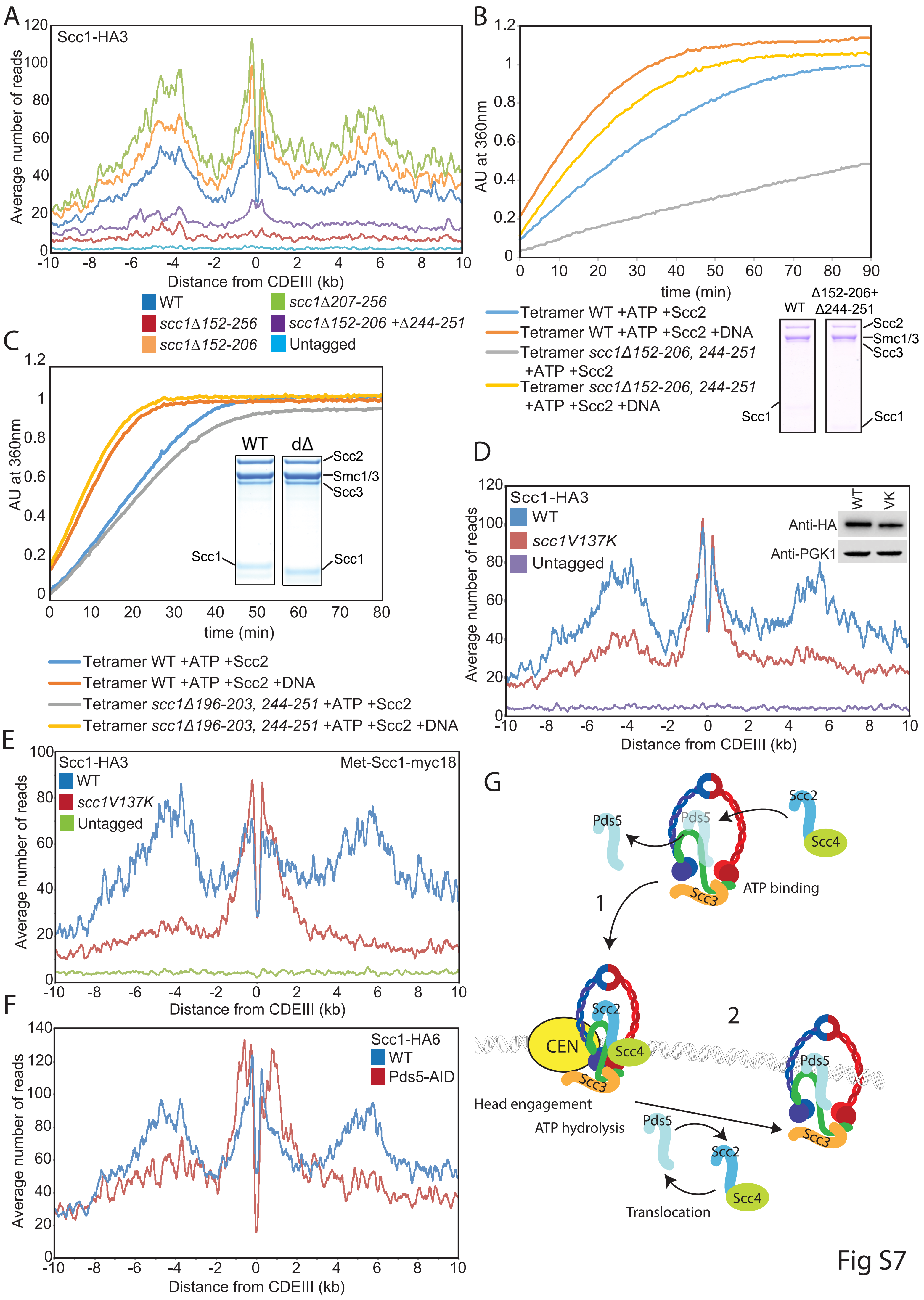

A
Scc1-HA3
Average number of reads
Distance from CDEIII (kb)
WT
scc1Δ152-256
scc1Δ152-206
scc1Δ207-256
scc1Δ152-206 +Δ244-251
Untagged
B
AU at 360nm
time (min)
Tetramer WT +ATP +Scc2
Tetramer WT +ATP +Scc2 +DNA
Tetramer scc1Δ152-206, 244-251 +ATP +Scc2
Tetramer scc1Δ152-206, 244-251 +ATP +Scc2 +DNA
WT
Δ152-206+ Δ244-251
Scc2
Smc1/3
Scc3
Scc1
C
AU at 360nm
time (min)
WT
dΔ
Scc2
Smc1/3
Scc3
Scc1
Tetramer WT +ATP +Scc2
Tetramer WT +ATP +Scc2 +DNA
Tetramer scc1Δ196-203, 244-251 +ATP +Scc2
Tetramer scc1Δ196-203, 244-251 +ATP +Scc2 +DNA
D
Scc1-HA3
WT
scc1V137K
Untagged
WT
VK
Anti-HA
Anti-PGK1
Average number of reads
Distance from CDEIII (kb)
E
Scc1-HA3
Met-Scc1-myc18
WT
scc1V137K
Untagged
Average number of reads
Distance from CDEIII (kb)
F
Scc1-HA6
WT
Pds5-AID
Average number of reads
Distance from CDEIII (kb)
G
Pds5
Scc2
Scc4
Scc3
ATP binding
1
2
CEN
Head engagement
ATP hydrolysis
Translocation


Fig S7

**S7 – Related to Figure 7**

**(A)** Average calibrated ChIP-seq profiles of HA-tagged WT and mutated Scc1 proteins in cycling cells at 25°C in the presence of untagged *SCC1* (K17184, K25896, K25995, K25997, K26660, K699). **(B)** Effect of *scc1Δ152-206 Δ244-251* on tetramer ATPase activity. A fraction of the mix was then analysed by Coomassie staining following SDS-PAGE. **(C)** Effect of *scc1Δ196-203 Δ244-251* on tetramer ATPase. **(D)** Average calibrated ChIP-seq profile comparing *scc1V137K*-HA3 to WT in the presence of the untagged endogenous Scc1 protein, in cycling cells. Protein levels of WT and *scc1V137K* were compared by Western blot with PGK1 as a loading control (K17184, K17190, K699). **(E)** Average calibrated ChIP-seq profiles of WT or V137K Scc1-HA3 in cells expressing endogenous *SCC1* under either the *MET3* promoter or the endogenous *SCC1* promoter. Cells were arrested in G1 (α factor) in the presence of methionine prior to release into methionine containing medium. Samples were taken at 60 min after release (K25222, K26248, K26251). **(F)** Average calibrated ChIP-seq profiles of Scc1-HA6 in the presence or absence of Pds5. Cells were arrested in G1 (α factor) prior to release for 90 min into auxin and nocodazole containing medium (K26270, K26277). **(G)** Two step model of cohesin loading onto DNA. Initially Scc2 displaces Pds5 from Scc1 and binding of ATP engages the SMC heads. Then ATP hydrolysis, stimulated by Scc2 and DNA, converts the cohesin ring to a state able to stably associate with DNA and translocate to neighbouring sequences. Once stably associated, repeated displacement of Pds5 by Scc2 drives the ATPase activity allowing for further translocation.

Table S1. Related to Figure 4.

| Scc2 Mutation | Viability |
|---|---|
| E416R | Viable |
| K432E | Viable |
| Q564R | Viable |
| E607K | Viable |
| N627H | Viable |
| E637K | Viable |
| R716L* | Viable |
| R716E* | Viable |
| S717L | Viable |
| S717L, K721E | **Lethal** |
| K721E | Viable |
| D730V | Viable |
| K755Q* | Viable |
| K755E* | Temperature sensitive at 35.5°C |
| D756A | Viable |
| A757L | Viable |
| R787T* | Viable |
| K788E | Viable |
| H789E | Viable |
| K788E, H789E | **Lethal** |
| D756A, K788A, H789A | Viable |
| S946P | Viable |
| L948F | Viable |
| D951Y | Viable |
| F1071P* | **Lethal** |
| R1073D | Viable |
| F1371H* | Viable |

* Residues mutated in CdLS

Table S2 – Yeast strains, related to STAR Methods section "Experimental Models: Organisms/Strains".

| Genotype | Identifier |
|---|---|
| *S. cerevisiae Mat a, W303 wildtype* (all other strains derived from this background) | K699 |
| *S. cerevisiae Mat a, scc2-45::NATMX4* | B443 |
| *S. cerevisiae Mat a, scc2-45::NATMX4: ura3::GAL1P-PDS5::URA3 (six copies)* | B1282 |
| *S. cerevisiae Mat a, ura3::GAL1P-PDS5::URA3 (six copies)* | B1289 |
| *S. cerevisiae Mat a, SMC3/SMC3-PK6::KANMX6, SCC4-TetR::HPHMX4/SCC4-TetR::HPHMX4, Alg9::TETO39_CaURA3::Mgs1/Alg9::TETO39_CaURA3::Mgs1* | B1612 |
| *S. cerevisiae Mat a, SCC3-PK6::KANMX6, SCC4-TetR::HPHMX4, Alg9::TETO39_CaURA3::Mgs1* | B1625 |
| *S. cerevisiae Mat a, SCC3-PK6::KANMX6, Alg9::TETO39_CaURA3::Mgs1* | B1627 |
| *S. cerevisiae Mat a, SCC1-PK6::TRP1, Alg9::TETO39_CaURA3::Mgs1* | B1635 |
| *S. cerevisiae Mat a, SMC3/SMC3-Pk6::KANMX6, Alg9::TETO39_CaURA3::Mgs1/Alg9::TETO39_CaURA3::Mgs1* | B1664 |
| *S. cerevisiae Mat a, PDS5-PK6::KANMX6, SCC4-TetR::HPHMX4, Alg9::TETO39_CaURA3::Mgs1* | B1665 |
| *S. cerevisiae Mat a, PDS5-PK6::KANMX6, Alg9::TETO39_CaURA3::Mgs1* | B1667 |
| *S. cerevisiae Mat a, SCC1-PK6::TRP1, SCC4-TetR::HPHMX4, Alg9::TETO39_CaURA3::Mgs1* | B1674 |
| *S. cerevisiae Mat a/alpha, Alg9::TETO39_CaURA3::Mgs1/Alg9::TETO39_CaURA3::Mgs1, SCC4-TetR::HPHMX4/SCC4-TetR::HPHMX4, SMC3/smc3(E1155Q)-PK6::NATMX4* | B1684 |
| *S. cerevisiae Mat a/alpha, Alg9::TETO39_CaURA3::Mgs1/Alg9::TETO39_CaURA3::Mgs1, SMC3/smc3(E1155Q)-PK6::NATMX4* | B1685 |
| *S. cerevisiae Mat a/alpha, SMC3/smc3(S1130R)-PK6::NATMX4, Alg9::TETO39_CaURA3::Mgs1/Alg9::TETO39_CaURA3::Mgs1* | B1748 |
| *S. cerevisiae Mat a/alpha, SMC3/smc3(S1130R)-PK6::NATMX4; SCC4-TetR::HPHMX4/SCC4-TetR::HPHMX4, Alg9::TETO39_CaURA3::Mgs1/Alg9::TETO39_CaURA3::Mgs1* | B1749 |
| *S. cerevisiae Mat a/alpha, SMC3/smc3(K38I)-PK6::NATMX4, Alg9::TETO39_CaURA3::Mgs1/Alg9::TETO39_CaURA3::Mgs1* | B1750 |
| *S. cerevisiae Mat a/alpha, SMC3/smc3(K38I)-PK6::NATMX4, SCC4-TetR::HPHMX4/SCC4-TetR::HPHMX4, Alg9::TETO39_CaURA3::Mgs1/Alg9::TETO39_CaURA3::Mgs1* | B1751 |
| *S. cerevisiae Mat a/alpha, SMC3/SMC3-PK6::KANMX6, Alg9::TETO39_CaURA3::Mgs1/Alg9::TETO39_CaURA3::Mgs1, SCC3-TetR::HPHMX4/SCC3-TetR::HPHMX4* | B1795 |
| *S. cerevisiae Mat a/alpha, SMC3/smc3(E1155Q)-PK6::NATMX4, Alg9::TETO39_CaURA3::Mgs1/Alg9::TETO39_CaURA3::Mgs1, SCC3-TetR::HPHMX4/SCC3-TetR::HphMX4* | B1796 |
| *S. cerevisiae Mat a/alpha, SMC3/smc3(S1130R)-PK6::NATMX4, Alg9::TETO39_CaURA3::Mgs1/Alg9::TETO39_CaURA3::Mgs1, SCC3-TetR::HPHMX4/SCC3-TetR::HPHMX4* | B1797 |
| *S. cerevisiae Mat a/alpha, SMC3/smc3(K38I)-PK6::NATMX4, Alg9::TETO39_CaURA3::Mgs1/Alg9::TETO39_CaURA3::Mgs1, SCC3-TetR::HPHMX4/SCC3-TetR::HPHMX4* | B1798 |
| *S. cerevisiae Mat a, leu2::SCC1-HA3::LEU2* | K17184 |
| *S. cerevisiae Mat a, leu2::scc1(V137K)-HA3::LEU2* | K17190 |
| *S. cerevisiae Mat a, trp1::SMC3-PK6::TRP1* | K17407 |

| | |
|---|---|
| *S. cerevisiae Mat a, trp1::smc3(E1155Q)-PK6::TRP1* | K17409 |
| *S. cerevisiae Mat a, SCC3-PK6::KANMX6* | K17438 |
| *S. cerevisiae Mat a, PDS5-PK6::KANMX6* | K19012 |
| *S. cerevisiae Mat a, SCC1-PK9::KANMX6, rad61Δ::HPHMX4, leu2::GAL1P-SIC1(9m)/HIS3P-GAL1/HIS3P-GAL2/GAL1P-GAL4::LEU2* | K20891 |
| *S. cerevisiae Mat a, SCC2-PK6::KANMX6* | K21388 |
| *S. cerevisiae Mat a, scc4Δ::HIS3, leu2::scc4-4::LEU2, SCC1-PK6::KANMX4* | K22001 |
| *S. cerevisiae Mat a, SCC1-PK6::KANMX4* | K22005 |
| *S. cerevisiae Mat a, SCC1-PK9::KANMX6, leu2::GAL1P-SIC1(9m)/HIS3P-GAL1/HIS3P-GAL2/GAL1P-GAL4::LEU2* | K22388 |
| *S. cerevisiae Mat a, SCC1-PK9::KANMX4, scc2-45::NATMX4* | K22390 |
| *C. glabrata Mat a, SCC1-PK9::NATMX4* | K23308 |
| *S. cerevisiae Mat a, smc1(D588E)::kiTRP1, scc4Δ::HIS3, YCplac33:scc4-4::NATMX4* | K23983 |
| *S. cerevisiae Mat a, SCC1-PK9::KANMX6, scc2-45::NATMX4, lys2::SCC2::HPHMX4::LYS2* | K24185 |
| *S. cerevisiae Mat a, SCC1-PK9::KANMX6, scc2-45::NATMX4, lys2::scc2(K788E, H789E)::HPHMX4::LYS2* | K24188 |
| *S. cerevisiae Mat a, scc2(E822K, L937F)::NATMX4, SCC1-PK6::KANMX4* | K24687 |
| *S. cerevisiae Mat a, scc2(E822K, L937F)::NATMX4, SCC1-PK6::KANMX4, scc4Δ::HIS3, leu2::scc4-4::LEU2* | K24744 |
| *S. cerevisiae Mat a, scc2(E822K, L937F)::NATMX4, SCC1-PK6::KANMX4, scc4Δ::HIS3* | K24745 |
| *S. cerevisiae Mat a, SCC1-PK6::KANMX6, scc4(F324A, K327D, K331D, K541D, K542D)::HIS3* | K25049 |
| *S. cerevisiae Mat a, scc2(E822K, L937F)-HA6::NATMX4* | K25053 |
| *S. cerevisiae Mat a, SCC2-HA6::NATMX4* | K25054 |
| *S. cerevisiae Mat a, SCC1-PK6::KANMX6, scc2(E822K, L937F)::NATMX4, chl4Δ::HPHMX4, scc4Δ::HIS3* | K25061 |
| *S. cerevisiae Mat a, lys2::SCC2-PK9::HPHMX4::LYS2* | K25185 |
| *S. cerevisiae Mat a, lys2::scc2(K788E, H789E)-PK9::HPHMX4::LYS2* | K25186 |
| *S. cerevisiae Mat a, scc1::METP-SCC1-myc18::TRP, SCC2-PK6::KANMX6* | K25222 |
| *S. cerevisiae Mat a, leu2::smc3(E1155Q)-HA3::LEU2, SCC1-PK6::KANMX6* | K25370 |
| *S. cerevisiae Mat alpha, leu2::smc3(E1155Q)-HA3::LEU2, SCC3-PK6::KANMX6* | K25373 |
| *S. cerevisiae Mat a, leu2::smc3(E1155Q)-HA3::LEU2, PDS5-PK6::KANMX6* | K25376 |
| *S. cerevisiae Mat alpha, scc2(E822K, L937F)-HA6::NATMX4, scc4Δ::HIS3, leu2::scc4-4::LEU2* | K25418 |
| *S. cerevisiae Mat a, PDS5-PK6::KANMX4, leu2::GAL1P-SIC1(9m)/HIS3P-GAL1/HIS3P-GAL2/GAL1P-GAL4::LEU2* | K25448 |
| *S. cerevisiae Mat a, leu2::smc3(E1155Q)-HA3::LEU2, SCC2-PK9::NATMX4* | K25467 |
| *C. glabrata Mat a, SCC1-HA3::NATMX4* | K25532 |
| *S. cerevisiae Mat a, SCC1-PK6::KANMX4, leu2::GAL1P-SIC1(9m)/HIS3P-GAL1/HIS3P-GAL2/GAL1P-GAL4::LEU2* | K25625 |
| *S. cerevisiae Mat a, SCC3-PK6::KANMX6, ura3::smc1(E1158Q)-myc9::URA3* | K25637 |
| *S. cerevisiae Mat a, SCC1-PK6::KANMX6, ura3::smc1(E1158Q)-myc9::URA3* | K25640 |
| *S. cerevisiae Mat a, SCC2-PK6::KANMX6, ura3::smc1(E1158Q)-myc9::URA3* | K25644 |
| *S. cerevisiae Mat a, PDS5-PK6::KANMX6, ura3::smc1(E1158Q)-myc9::URA3* | K25652 |
| *S. cerevisiae Mat a, leu2::scc1(Δ152-256)-HA3::LEU2* | K25896 |
| *S. cerevisiae Mat a, scc2(E822K, L937F)::NATMX4, PDS5-PK6::KANMX4, leu2::GAL1P-SIC1(9m)/HIS3P-GAL1/HIS3P-GAL2/GAL1P-GAL4::LEU2* | K25988 |
| *S. cerevisiae Mat a, leu2::scc1(Δ152-206)-HA3::LEU2* | K25995 |
| *S. cerevisiae Mat a, leu2::scc1(Δ207-256)-HA3::LEU2* | K25997 |

| | |
|---|---|
| *S. cerevisiae Mat a, PDS5-PK6::KANMX4, leu2::GAL1P-SIC1(9m)/HIS3P-GAL1/HIS3P-GAL2/GAL1P-GAL4::LEU2* | K25999 |
| *S. cerevisiae Mat a, scc2(E822K, L937F)::NATMX4, SCC1-PK6::KANMX4, leu2::GAL1P-SIC1(9m)/HIS3P-GAL1/HIS3P-GAL2/GAL1P-GAL4::LEU2* | K26244 |
| *S. cerevisiae Mat a, scc1::MET3P-SCC1-MYC18::TRP1, SCC2-PK6::KANMX4, leu2::SCC1-HA3::LEU2* | K26248 |
| *S. cerevisiae Mat a scc1::MET3P-SCC1-MYC18::TRP1, SCC2-PK6::KANMX4, leu2::scc1(V137K)-HA3::LEU2* | K26251 |
| *S. cerevisiae Mat a, SCC1-HA6::HIS3, ura3::ADH1P-OsTIR1-9MYC::URA3, PDS5-PK3-AID::KANMX4, leu2::GAL1P-SIC1(9m)/HIS3P-GAL1/HIS3P-GAL2/GAL1P-GAL4::LEU2* | K26270 |
| *S. cerevisiae Mat a, SCC2-HA6::NATMX4, ura3::ADH1P-OsTIR1-9MYC::URA3, PDS5-PK3-AID::KANMX4, leu2::GAL1P-SIC1(9m)/HIS3P-GAL1/HIS3P-GAL2/GAL1P-GAL4::LEU2* | K26273 |
| *S. cerevisiae Mat a, SCC2-HA6::NATMX4, leu2::GAL1P-SIC1(9m)/HIS3P-GAL1/HIS3P-GAL2/GAL1P-GAL4::LEU2* | K26274 |
| *S. cerevisiae Mat a, SCC1-HA6::HIS3, leu2::GAL1P-SIC1(9m)/HIS3P-GAL1/HIS3P-GAL2/GAL1P-GAL4::LEU2* | K26277 |
| *S. cerevisiae Mat a, SCC1-PK6::KANMX4, scc3(K404E)-HA3::HIS3, rad61::HPHMX4, leu2::GAL1P-SIC1(9m)/HIS3P-GAL1/HIS3P-GAL2/GAL1P-GAL4::LEU2* | K26281 |
| *S. cerevisiae Mat a, SCC1-PK6::KANMX4, scc3(K404E)-HA3::HIS3, leu2::GAL1P-SIC1(9m)/HIS3P-GAL1/HIS3P-GAL2/GAL1P-GAL4::LEU2* | K26286 |
| *S. cerevisiae Mat a, SCC1-PK6::KANMX4, rad61::HPHMX4, leu2::GAL1P-SIC1(9m)/HIS3P-GAL1/HIS3P-GAL2/GAL1P-GAL4::LEU2* | K26288 |
| *S. cerevisiae Mat a, SCC1-PK6::KANMX4, leu2::GAL1P-SIC1(9m)/HIS3P-GAL1/HIS3P-GAL2/GAL1P-GAL4::LEU2* | K26289 |
| *S. cerevisiae Mat a, SCC1-PK6::KANMX4, leu2::GAL1P-SIC1(9m)/HIS3P-GAL1/HIS3P-GAL2/GAL1P-GAL4::LEU2* | K26292 |
| *S. cerevisiae Mat a, leu2::scc1(Δ164-172, Δ207-256)-HA3::LEU2* | K26360 |
| *S. cerevisiae Mat a, leu2::scc1(Δ152-219)-HA3::LEU2* | K26361 |
| *S. cerevisiae Mat alpha, leu2::scc1(Δ152-206, Δ244-251)-HA3::LEU2* | K26362 |
| *Mat a, leu2::scc1(Δ152-206 + Δ244-251)-HA3::LEU2* | K26660 |
| *S. cerevisiae Mat a/alpha, lys2::SCC2-GFP::HphMX::LYS2, MTW1-RFP::ADE2* | K26872 |
| *S. cerevisiae Mat a/alpha, lys2::scc2(K788E, H789E)-GFP::HPHMX4::LYS2, MTW1-RFP::ADE2* | K26875 |
| *S. cerevisiae Mat alpha, leu2::scc1(Δ196-203, Δ244-251, Δ281-288)-HA3::LEU2* | K26992 |
| *S. cerevisiae Mat alpha, leu2::scc1(Δ196-203, Δ244-251)-HA3::LEU2* | K27148 |
